# Microscale Architectures for Intelligent Soft Robotics: From Functional Microneedles to Biointegrated Wearable Systems

**DOI:** 10.1007/s40820-025-02026-2

**Published:** 2026-01-05

**Authors:** Xin Li, Ran Xu, Chenchen Xie, Zhixing Ge, Bingbing Gao, Chwee Teck Lim

**Affiliations:** 1 College of Biotechnology and Pharmaceutical Engineering, Nanjing Tech University, Nanjing, 211816 People’s Republic of China; 2 School of Pharmaceutical Sciences, Nanjing Tech University, Nanjing, 211816 People’s Republic of China; 3 Institute for Health Innovation and Technology (iHealthtech), National University of Singapore, Singapore, 117599 Singapore; 4 Department of Biomedical Engineering, National University of Singapore, Singapore, 117583 Singapore; 5 Mechanobiology Institute, National University of Singapore, Singapore, 117411 Singapore

**Keywords:** Soft robotics, Microneedle arrays, 4D-printed hydrogels, Stimuli-responsive materials

## Abstract

Soft robots, characterized by compliance, adaptability, and multimodal responsiveness, represent a rapidly advancing frontier in biomedical applications, wearable technologies, and environmental exploration. This review summarizes recent progress in soft robotics with a focus on material innovation, structural design, functional integration, and intelligent responsiveness. Emphasis is placed on the development of bioinspired and stimuli-responsive materials, the construction of modular and reconfigurable architectures, and the integration of actuation, sensing, and energy systems. Microneedle array-based soft robots and hydrogel-based 4D-printed systems are introduced as representative platforms for drug delivery, wound healing, and environmental monitoring. Key challenges, including limited durability, power autonomy, and multifunctional synergy, are critically analyzed in relation to practical operation and long-term reliability. Future directions involve the convergence of self-healing materials, intelligent control algorithms, and multiscale integration strategies to achieve enhanced adaptability and clinical translation. This review provides a comprehensive overview of the interdisciplinary development of next-generation soft robots that bridge materials science, biomedical engineering, and intelligent systems, paving the way toward real-world applications.

**Supplementary Information:**

The online version contains supplementary material available at 10.1007/s40820-025-02026-2.

## Highlights

- Comprehensive perspective on soft robotic systems integrating material innovation, structural design, functional synergy, and intelligent control across biomedical and environmental applications.
- Representative platforms including microneedle array-based soft robots and 4D-printed hydrogel systems are analyzed to demonstrate programmable actuation, sensing, and therapeutic functions.
- Critical challenges and future directions are outlined, emphasizing modular standardization, self-healing materials, and data-driven control strategies for next-generation adaptive soft robots.

**Supplementary Information:**

The online version contains supplementary material available at 10.1007/s40820-025-02026-2.

## Graphical abstract

**Figure:**
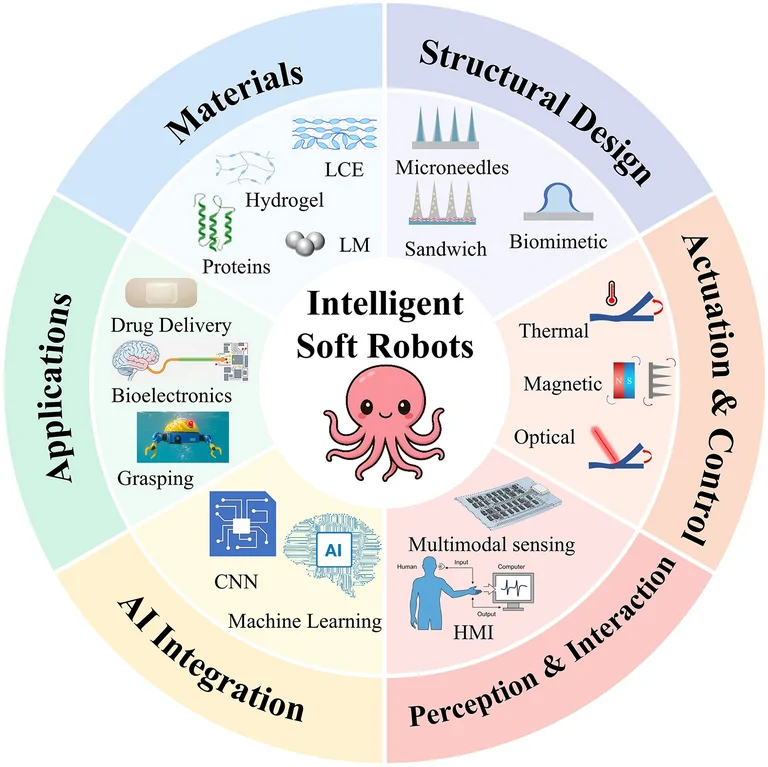

## Introduction

**Fig. 1:**
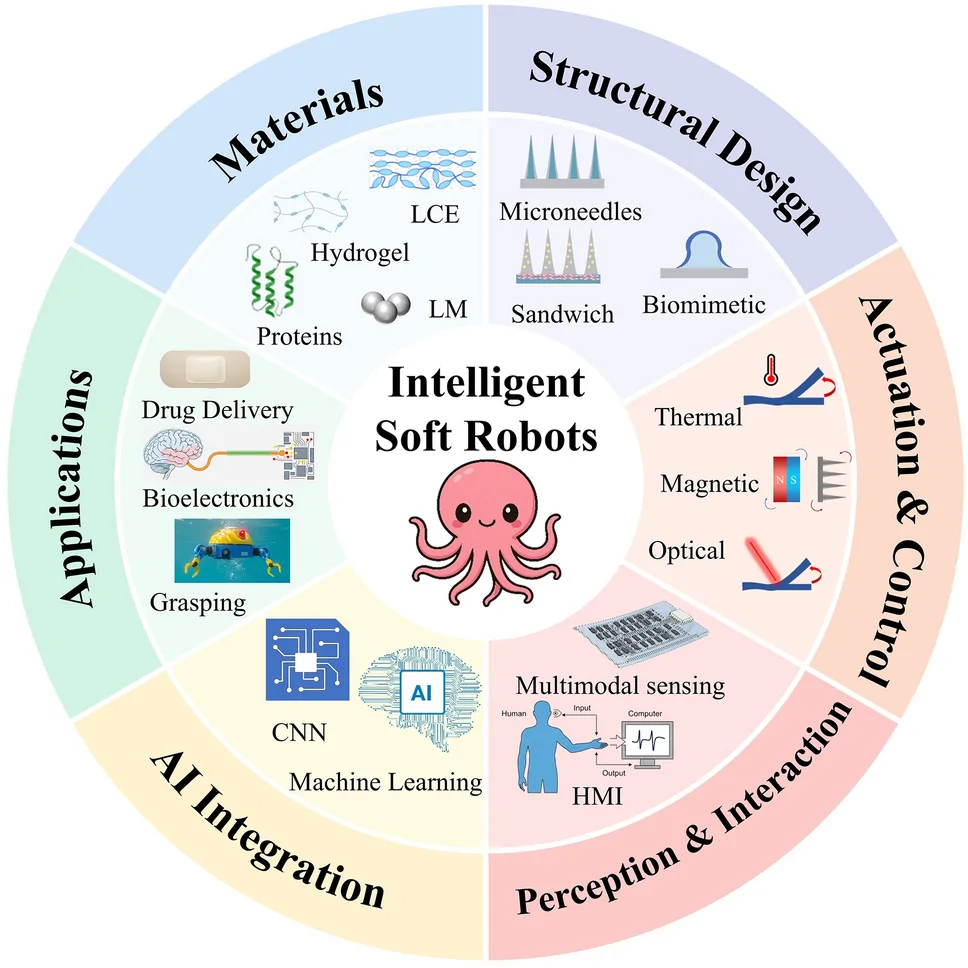
Microneedle-integrated soft robotic systems enabling sensing, actuation, and therapeutic applications across biomedical interfaces

**Fig. 2:**
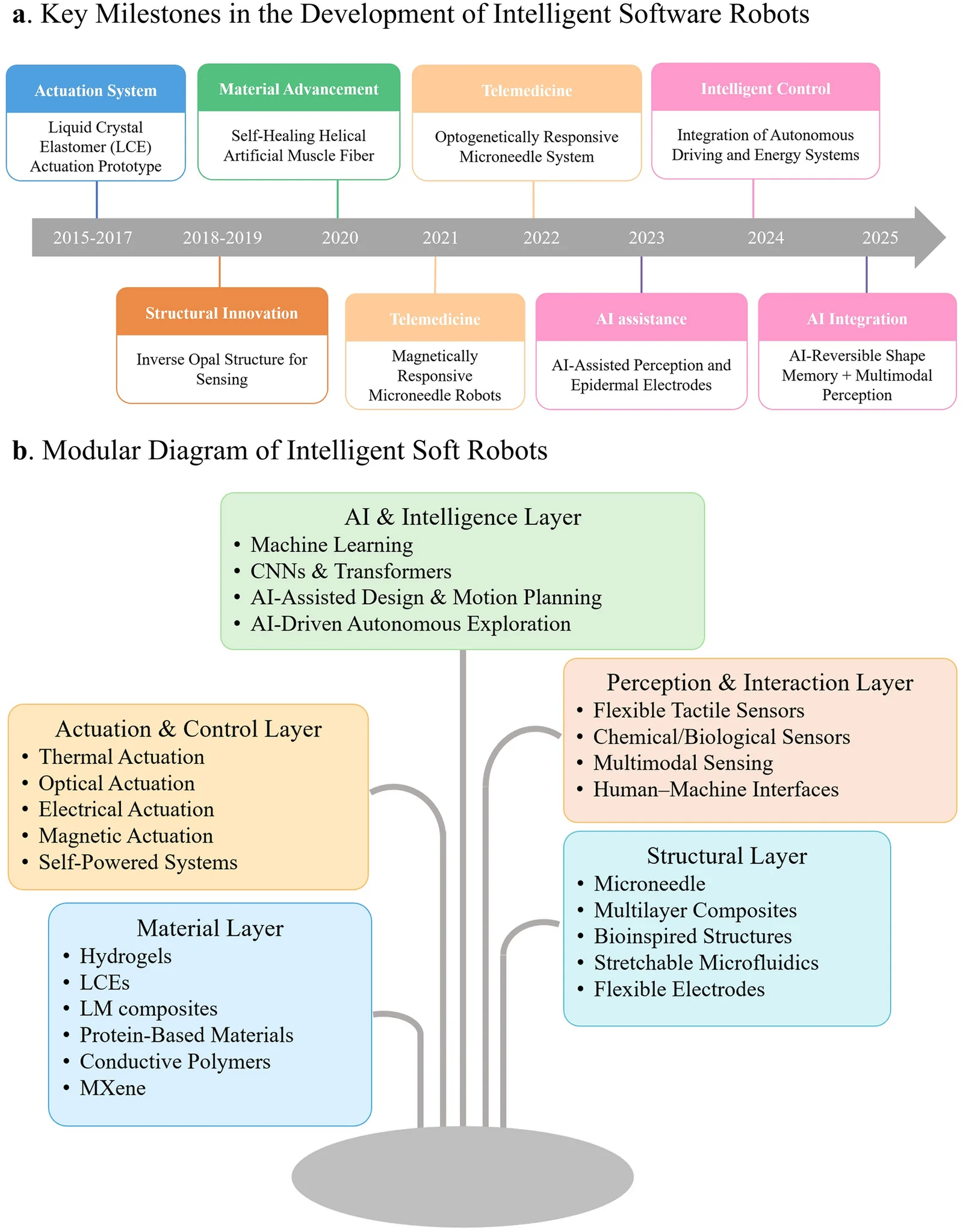
**a** Timeline of key technological milestones in the development of intelligent soft robots from 2015 to 2025. The diagram highlights advances in materials, structural innovations, actuation mechanisms, AI-assisted sensing, and system-level integration. **b** Modular schematic of intelligent soft robots illustrating the hierarchical architecture and functional interactions between five core subsystems. The material layer provides the physical foundation, determining flexibility, biocompatibility, and responsiveness. The structural layer builds on this by defining robot morphology and adaptability through advanced structural design. The actuation and control layer enables dynamic movements via multimodal stimuli. The perception and interaction layer facilitates closed-loop sensing and real-time environmental feedback. At the top, the AI and intelligence layer governs system-level autonomy through learning-based decision-making, optimization, and task execution

Soft robots represent a new paradigm in engineering [1–4], where functionality is achieved not through rigid mechanical assemblies but through compliant materials, stimuli‐responsive architectures, and distributed control frameworks [5–9] (Fig. 1). By harnessing the inherent deformability of soft matter and adopting bioinspired strategies, these systems exhibit remarkable adaptability, environmental compatibility, and multimodal responsiveness [10, 11]. Their capability for large, reversible deformations while maintaining safe interactions with biological interfaces allows operation under unstructured conditions, driving broad interest across biomedical, wearable, and environmental domains [12, 13]. Early investigations centered on passive materials and single‐mode deformation [14, 15]; however, the integration of sensing, actuation, and logic within soft matrices has enabled the transition toward intelligent machines capable of environmental adaptation and programmable behaviors [16–19]. This technological trajectory illustrates the shift from isolated component design to holistic, feedback‐driven system integration [20] (Fig. 2a).

To support such functional complexity, modular architecture has emerged as a core strategy in soft system design. Rather than monolithic bodies, contemporary soft robots are assembled from interoperable modules dedicated to structure, actuation, and control, sensing and interaction, energy management, and intelligence. Figure 2b presents this hierarchical framework, progressing from material and structural foundations to actuation/control, perception/interaction, and finally an AI‐driven intelligence layer. While modularity enhances scalability and functional reconfigurability, it also introduces critical challenges in cross‐module compatibility and standardization. Interfaces between actuation modules and energy modules often lack unified mechanical and electrical coupling protocols, restricting seamless assembly and reproducibility. Efforts toward soft‐bus architectures, wireless signal transmission, and adaptive interface protocols are emerging [21, 22], yet universal industrial or academic standards remain under development. Establishing such standardized interconnects will be essential for scalable manufacturing, data interoperability, and translational deployment of modular soft robotic systems.

At the foundation of these architectures lie adaptive materials capable of coupling mechanical compliance with functional responsiveness. Elastomers, hydrogels, protein‐based polymers, and liquid metals enable tunable stiffness, reversible deformation, and multifield responses under mechanical, thermal, optical, and chemical stimuli [23, 24]. These materials act as active transducers—capable of sensing, actuating, and self‐healing—to emulate biological tissue dynamics across multiple length scales [25–27]. Equally important, structural design dictates motion pathways, force transmission, and overall stability [28–31]. Bioinspired geometries such as coiled fibers, folded membranes, lattice scaffolds, and hierarchical composites have produced architectures that undergo anisotropic deformation, gradient stiffening, and geometry‐encoded locking. Other emerging strategies, including braided architectures [32, 33], offer enhanced mechanical robustness and programmable compliance, enriching the design space for adaptive motion. The integration of gradient mechanics and internal patterning further enables programmable stiffness and spatially distributed functionality [34–38], transforming structural design into an active contributor to system intelligence.

Advances in fabrication technologies have expanded the complexity and responsiveness of soft robotic systems. In particular, 4D printing—the temporal extension of 3D printing—introduces time as the “fourth dimension,” whereby printed architectures are designed to evolve their shapes or properties in response to external stimuli [20, 39–42]. Unlike conventional additive manufacturing, 4D‐printed hydrogel systems incorporate preprogrammed time‐dependent transformations such as swelling, shrinking, or phase transition, producing dynamic architectures that autonomously change form or function in real time. By integrating spatially resolved material placement with sequential deformation encoding, these systems bridge the gap between static mechanical design and living matter, enabling adaptive morphing, reversible actuation, and environment‐responsive control. Such capability marks a major step toward creating self‐regulated and reconfigurable soft robots for biomedical and environmental applications.

Despite these advances, key challenges remain. Long‐term mechanical durability, power autonomy, closed‐loop control, and clinical compatibility still limit the transition of soft robotic platforms from laboratory demonstrations to practical use [43–45]. Moreover, the absence of universal modular standards and limited scalability hinder reproducibility and system integration. This review therefore provides a detailed examination of soft robotics from a material–structure–function integration perspective, focusing on microneedle (MN) platforms [46–51], 4D‐printed hydrogel devices [52–58], and composite architectures [59–64], which collectively represent leading directions toward adaptive and intelligent soft systems [65, 66]. By summarizing representative breakthroughs, clarifying conceptual definitions, and analyzing existing challenges, this work aims to outline a coherent framework for designing the next generation of soft robots that combine structural versatility, functional intelligence, and seamless interaction across biomedical and environmental interfaces.

## Structure and Materials of Soft Robots

**Fig. 3:**
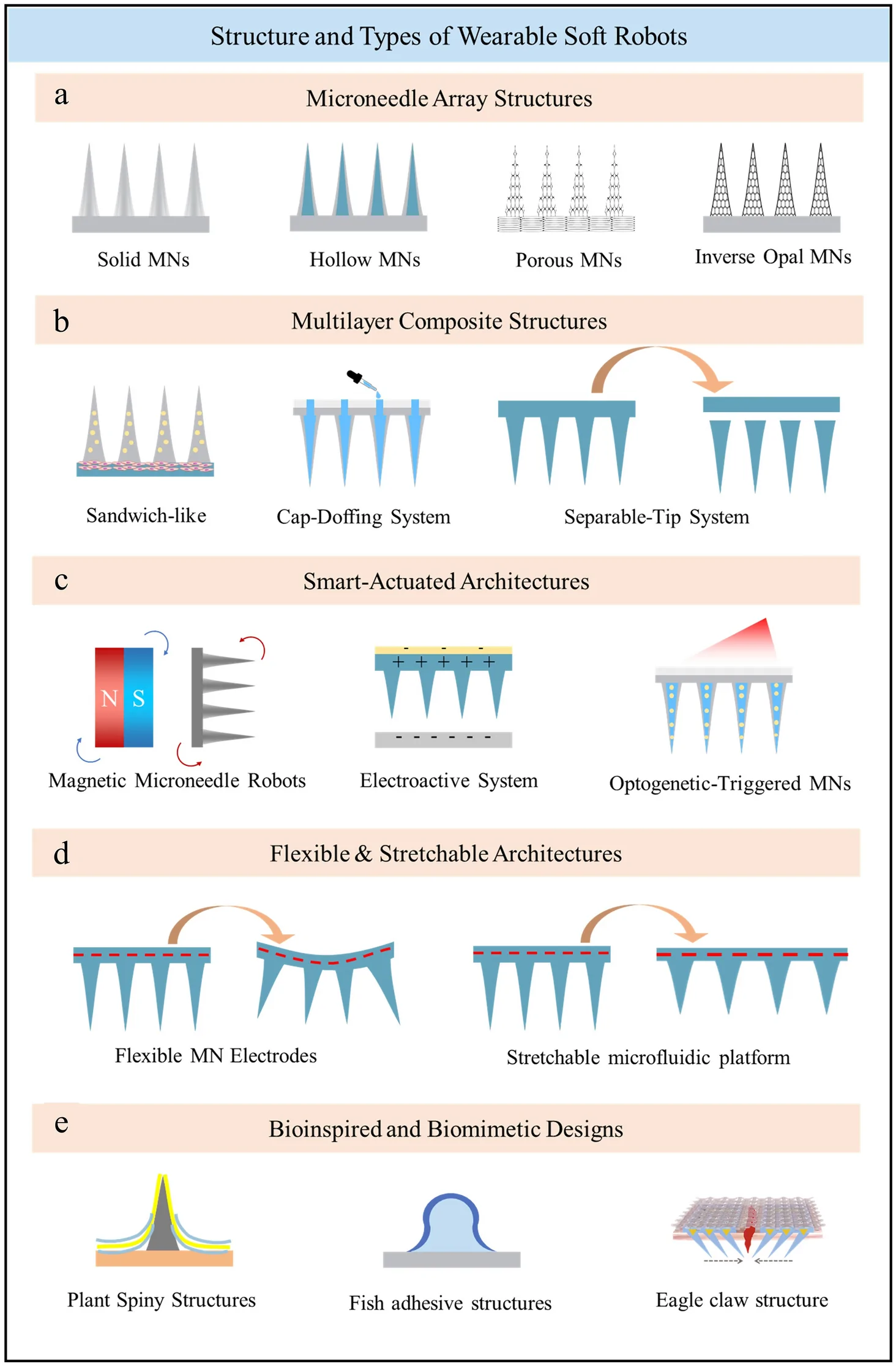
Structural diversity and types of wearable soft robots.** a** Microneedle array structures, **b** multilayer composite structures, **c** smart-actuated architectures, **d** flexible and stretchable platforms, and **e** bioinspired and biomimetic designs, revealing a broad spectrum of approaches for developing advanced soft robotic systems

Soft robots integrate functionally diverse structural forms, such as solid, hollow, porous, and cavity-tunable microneedles [67–73] (Fig. 3), with advanced material systems, including hydrogels, elastomers, and conductive composites [74–77] (Table 2). Through multilayer architectures [78–82] and actuation [83], these systems achieve precise control over drug delivery, sensing, and mechanical interactions. Bioinspired designs and flexible substrates further increase the adaptability and biocompatibility of these materials [84–86], supporting their application in dynamic physiological environments.

### Structure

#### Microneedle Array Structures

Microneedle array structures are emerging as a versatile class of microstructured interfaces that significantly advance the perception, actuation, and functional integration of soft robotic systems (Table 1). Solid MN arrays, exemplified by silica gel-based microarray tactile sensors, endow soft robots with refined perception of object shape, size, and surface texture, enabling high-resolution tactile feedback and intelligent recognition when coupled with machine learning algorithms [87]. Moreover, conical solid MNs reinforced with materials such as carbon nanotubes or hydrogels exhibit excellent mechanical robustness and adaptability, offering structural design principles for integrating sensing and actuation functions within flexible robotic platforms [88] (Table 2).

**Table 1:** Quantitative comparison of representative microneedle (MN) array structures and their key performance metrics

MN type	Representative materials/fabrication	Mechanical strength (MPa)	Response time/functional latency	Drug loading efficiency or flow rate	Distinctive functionality	Potential soft-robotics application	Refs
Solid MNs	PDMS, CNT–hydrogel composites via replica molding	2–5 (compressive)	~ 10 ms (mechanical)	–	High stiffness, reliable tactile transmission	Tactile sensing arrays, pressure-mapping interfaces	[87, 88]
Hollow MNs	PI/PVDF-TrFE + SWCNT with Ni–Au plating	0.8–1.5 (axial)	< 1 s (fluid)	1–10 µL min⁻^1^ (glucose solution)	Bidirectional flow, real-time sampling	Microfluidic actuation, biofluidic control, drug infusion	[89, 90]
Porous MNs	Mesoporous SiO₂ core + chitosan hydrogel sealant	0.3–0.8	2–5 min (pH/glucose-triggered)	20%–35%	Self-regulated release, diffusion-controlled permeability	Chemical sensing, stimuli-responsive drug delivery	[91]
Inverse-opal MNs	Colloidal-crystal-templated hydrogel/silica composite	0.2–0.6	< 1 s (optical)	10%–20%	Photonic enhancement, ≈3 × fluorescence gain	Optical biosensing, real-time diagnostic readout	[92]

**Table 2:** Summary of the characteristics of intelligent soft robotics

Material type	Structure/form and application examples	Advantages	Disadvantages	Functional properties	Refs
Hydrogel	All-hydrogel sensors, composite hydrogels, PNIPAM conductive networks	Softness, fast response, biocompatibility, printable, self-healing, controlled release	Easy dehydration, low mechanical strength, poor environmental stability	Stretchability up to 3734%, conductivity 3.41 S m⁻^1^	[93–98]
Liquid crystal elastomers (LCEs)	Hollow LCE fibers, artificial muscles, programmable origami, helical actuators	Fast actuation, programmable deformation, high driving force, long cycle life	Complex fabrication, limited response to temperature/light, low electroactuation efficiency	Strain rate up to 1522%/s, power density 7.03 kW kg⁻^1^	[99–102]
Liquid metal composites	LM hydrogels, LM-based TENGs, LM conductive networks, self-healing electronic skins	High conductivity, stretchable, soft, self-healing, energy harvesting, intelligent sensing	High cost, high density, interfacial challenges, potential toxicity	Conductivity up to 9 × 10⁶ S m⁻^1^, 100% self-healing efficiency, > 90% transparency	[103–109]
Protein-based materials	Spider silk, silk/gelatin/keratin hydrogels, TENGs, artificial muscles	Good biodegradability, diverse stimulus response, rapid thermohumidity responsiveness, eco-friendly	Limited mechanical strength, environmentally dependent performance, nonnegligible cost	Actuation stress up to 18 MPa, TENG output voltage up to 2.1 kV	[110–115]
MXene composites	Multiresponsive actuators, electrochemical sensors, flexible energy systems	Multifield response, high conductivity, strong mechanical properties, suitable for energy/storage/sensing	Poor oxidation stability, low dispersion, insufficient flexibility	Specific capacitance up to 410 F g⁻^1^ (at 2 mV s⁻^1^), areal capacitance ~ 360 mF cm⁻^2^, energy density ≈ 36 Wh kg⁻^1^, and stable operation over > 10,000 cycles also exhibit capacitive and pseudocapacitive behavior, low-temperature stability (− 20 °C), and high-frequency response	[116–118]
Conductive polymers	Self-healing hydrogels, flexible electrodes	Good processability, excellent flexibility and conductivity, high transparency	Poor mechanical robustness, limited environmental stability	Transparency up to 93%, conductivity 4.85 S m⁻^1^	[106]
LCE-based composite structures	Liquid metal–spider silk protein–LCE 4D-printed actuators	Enables remote/programmed shape transformation + strain sensing, high integration	Sensitive to printing parameters, difficult interfacial control	Regional response, photothermal responsiveness, impedance-strain dual sensing	[119]

Hollow MNs extend the functionality of these systems by combining biosensing, microfluidic regulation, and targeted delivery within a single microarchitecture [89, 90]. Flexible hollow microfiber arrays fabricated from nanocomposite materials with metallic coatings enable continuous biofluid extraction and electrochemical biosensing, laying the foundation for conformable, self-diagnostic robotic skins. In parallel, adjustable hollow polymer MNs with tunable cavity volumes achieve precise control over release kinetics, suggesting potential applications as biofluidic actuators and chemical delivery modules in soft robotic devices, thereby bridging biomedical and robotic domains.

Beyond solid and hollow configurations, porous, inverse opal, and hybrid MNs open new frontiers for adaptive and intelligent robotic functionalities. Porous MNs constructed from mesoporous silica and sealed with chitosan hydrogels exhibit glucose-responsive, self-regulated insulin release, offering a paradigm for stimuli-responsive and self-adaptive soft robotic systems [71, 91]. Inverse opal MNs, derived from colloidal crystal templating, possess highly ordered porous networks that amplify fluorescence and enhance biofluid extraction, enabling real-time biosensing and high-fidelity diagnostic feedback [92]. Meanwhile, hybrid MNs that integrate multiple structural motifs and material components are poised to unify sensing, actuation, and controlled delivery within a single soft robotic platform—paving the way toward autonomous, multifunctional, and biointegrated robotic interfaces.

#### Multilayer Composite Structures

Multilayer composite structures have emerged as a vital design paradigm in soft robotics, enabling the integration of diverse functionalities within compact and flexible architectures. Sandwich-like systems exemplify this approach through the assembly of layered components that synergistically perform distinct yet complementary tasks [120]. A representative case is the conductive microneedle patch for myocardial infarction therapy, comprising a drug-loaded microneedle base for localized release, a middle carbon nanotube (CNT) layer for electrical conduction, and a GelMA hydrogel scaffold supporting induced cardiomyocytes. This hierarchical configuration enables simultaneous drug delivery, electrical stimulation, and cell integration, demonstrating how multilayer structural design can achieve biocompatible, multifunctional, and adaptive interfaces that meet the complex demands of soft robotic operation and biomedical repair.

The “Cap-Doffing System” represents another sophisticated multilayer concept that introduces self-regulation through dynamic molecular gating [91]. In this architecture, mesoporous silica microneedles capped with enzyme-loaded chitosan hydrogels act as stimuli-responsive valves. Changes in glucose concentration trigger enzymatic reactions that cause reversible swelling or deswelling of the hydrogel cap, achieving on-demand, repeatable, and reversible release of encapsulated substances. This dynamic control mechanism mirrors the adaptive behaviors desired in soft robots, enabling responsive substance release, intelligent channel regulation, and potential integration into chemical sensing, self-healing materials, or bioinspired actuation systems capable of precise environmental interaction.

The separable-tip system exemplifies an advanced design philosophy for safe and targeted in vivo operation of microrobotic systems [121]. In this configuration, functional and control units are decoupled through a detachable interface—such as in magnetically guided microneedle robots designed for oral delivery of macromolecular drugs. The device features a magnetic base, separable connector, and drug-loaded tip fabricated via a modular multistage 3D strategy. After reaching the intestine, the magnetic base facilitates precise positioning and penetration, after which the degradable connector detaches, leaving the tip embedded for sustained drug release while the base is safely excreted. This architecture not only enhances bioavailability and long-term efficacy but also establishes a biosecure and programmable model for future intelligent soft robots designed for minimally invasive therapeutic delivery.

#### Smart-Actuated Architectures

Smart-actuated architectures have become a pivotal frontier in soft robotics, enabling remote, programmable, and biologically integrated functionalities through the synergistic design of responsive microneedle (MN) systems. Magnetically driven MN robots exemplify this progress, achieving noncontact manipulation and targeted intervention under external magnetic fields [122–124]. A representative design features a modular, multistage 3D-printed robot composed of a magnetic base, detachable connector, and drug-loaded tip. Guided by a magnetic field after oral administration, the robot precisely penetrates the intestinal wall to deliver macromolecular therapeutics such as insulin, followed by autonomous detachment and excretion of the magnetic component, thereby enhancing drug bioavailability and biosafety. Moreover, layered magnetic MN array robots have demonstrated precise tissue cutting for organoid-on-a-chip fabrication, underscoring their potential for minimally invasive surgery and advanced bioengineering. These examples highlight how magnetic control endows soft robotic systems with superior locomotion precision, multifunctional integration, and clinical translation potential.

Electroactive systems further advance the sensory intelligence of soft robots by converting electrical and mechanical stimuli bidirectionally, allowing real-time environmental perception and adaptive responses [88]. A representative microneedle tactile sensor based on silicone composites operates via triboelectric and electrostatic induction principles, translating shape, size, and texture cues into electrical signals for intelligent object recognition. This electroactive tactile system, when coupled with machine learning, achieves high-fidelity recognition of curved and deformable surfaces, effectively simulating the tactile sensitivity of biological skin. Such strategies not only provide new routes for material–structure co-design but also lay the technological foundation for autonomous, multimodal soft robots with refined environmental interactivity.

Optogenetically triggered MN systems represent an emerging paradigm that merges optogenetic precision with microneedle delivery to achieve spatiotemporally resolved biological control [125]. A triboelectric-responsive MN platform designed for intervertebral disk degeneration therapy exemplifies this strategy: optogenetically engineered extracellular vesicles (EVs) loaded within the microneedle array are released in a controlled manner through triboelectric stimulation, enabling optical regulation of intracellular DNA-sensing pathways associated with inflammation in degenerative cells. By integrating optogenetic actuation with microstructured interfaces, these systems provide light-controllable, noninvasive pathways for manipulating cellular behavior and biochemical signaling. This approach opens transformative opportunities for next-generation biorobots capable of intelligent diagnosis, precision therapy, and dynamic integration with living tissues.

#### Flexible and Stretchable Architectures

Flexible microneedle (MN) electrodes have emerged as pivotal components in the evolution of soft robotic systems, enabling seamless, conformable electrical interfaces with biological tissues for biosensing, neural modulation, and tissue engineering applications. These electrodes combine minimally invasive penetration with high mechanical compliance, allowing stable signal transduction across irregular or dynamic surfaces. Hollow MN arrays constructed from soft conductive microfibers via nickel–gold plating have been reported as stretchable microfluidic biosensing patches integrating flexible electrochemical sensors, capable of interstitial fluid sampling and precise biomolecular detection under deformation conditions. Such systems exemplify the synergistic integration of conductivity, flexibility, and biointerface conformity, paving the way for next-generation wearable diagnostic platforms. Furthermore, an inductively integrated conductive MN patch designed for myocardial infarction therapy employs aligned carbon nanotube (CNT) intermediate layers to form efficient electrical conduction pathways, imparting both flexibility and high conductivity to the patch. This configuration enables electrophysiological signal transmission and promotes cell activity and tissue regeneration. Collectively, these flexible MN electrodes—combining conductive materials with compliant substrates—minimize tissue damage, enhance signal stability, and improve long-term wearability, offering essential technological support for soft robotic systems in chronic monitoring, electrical stimulation, and therapeutic applications [87, 90, 126–128].

Stretchable microfluidic platforms represent another critical advancement in soft bioelectronics, offering deformable, skin-conformal systems that sustain both fluidic and electrical functionality under strain [63, 129]. By integrating microfluidic channels into elastomeric matrices, these platforms enable stable adhesion to curved or moving biological surfaces while maintaining precise liquid transport and signal fidelity. A representative stretchable biosensing patch featuring an array of hollow MNs composed of flexible microfibers integrates a microfluidic sampling module, an electrochemical biosensor, and a flexible substrate, forming a closed-loop analytical system capable of minimally invasive interstitial fluid collection and real-time biomarker detection, such as glucose monitoring. The negative pressure-driven sampling mechanism, coupled with the inherent elasticity of the substrate, ensures durable, user-friendly, and irritation-free operation. This design paradigm highlights the potential of integrating softness, multifunctionality, and precision into next-generation wearable point-of-care testing (POCT) devices, substantially broadening the role of soft robotic platforms in biofluidic sensing, smart diagnostics, and personalized healthcare [130].

The integration of flexible MN electrodes and stretchable microfluidic platforms thus defines a transformative framework for soft robotic systems. These hybrid architectures bridge the gap between mechanical adaptability and electrical or biochemical performance, creating robust, long-term biointerfaces capable of real-time physiological monitoring, targeted therapy, and closed-loop control. By harmonizing electronic conductivity, fluidic transport, and tissue compliance, such systems embody the next frontier of intelligent, body-conformal soft robots—offering unprecedented opportunities in biosensing, neural engineering, and regenerative medicine.

#### Bioinspired and Biomimetic Design

Bioinspired structural designs derived from natural organisms offer powerful strategies to enhance mechanical adaptability, adhesion, and long-term biointegration in soft robotic systems. Plant-thorn-inspired microneedle architectures, featuring tapered and backward-hooked geometries, enable secure yet minimally invasive anchoring at dynamic tissue interfaces [131]. Such designs have been translated into skin-integrated stretchable silicon microneedle electrodes (SSMEs) that combine silicon microneedle arrays with polyimide semi-encapsulation, achieving superior mechanical stability, fatigue resistance, and over 36% stretchability. These features ensure reliable electromyography (EMG) signal acquisition during motion while maintaining excellent biocompatibility and comfort.106 By mimicking the structural logic of natural spines, these microneedle interfaces achieve precise mechanical interlocking and robust conformal contact, providing a blueprint for high-fidelity, long-lasting human–machine interfaces in wearable bioelectronics and soft robotics.

In parallel, aquatic and avian organisms have inspired novel adhesion and anchoring mechanisms with unprecedented efficiency. Fish-like adhesion systems, modeled after the lamellar suction disks and microspines of climbing fish, integrate flexible lamellae for wet adhesion and stiff epidermal spines for directional friction, allowing strong attachment and agile sliding on moist or irregular substrates [132]. Extending this principle to terrestrial conditions, eagle-claw-inspired microhooks and microneedles replicate the curvature, stiffness gradient, and anisotropic grasping mechanics of avian talons, enabling stable, reversible anchoring with minimal tissue irritation [133, 134]. These nature-derived architectures collectively demonstrate how hierarchical structural design—combining softness, rigidity, and directional mechanics—can endow soft robotic systems with adaptive interfacial behaviors, secure biointegration, and enhanced operational stability across complex environments.

### Materials

#### Hydrogel-Based Materials for Soft Robots

Hydrogels have emerged as a pivotal materials platform for next-generation soft robotics and biomedical microdevices owing to their high water contents, tunable mechanics, and biocompatibility [93]. Recent advances have focused on enhancing the stimulus responsiveness of hydrogels to precisely regulate drug diffusion kinetics and actuation behavior in microneedle (MN)-based systems. Thermo-, pH-, and photo-responsive networks can dynamically alter mesh size and permeability under external stimuli, enabling programmable drug release synchronized with robotic motion or physiological cues. For instance, PNIPAM-based or silk protein–reinforced hydrogels demonstrate rapid volumetric transitions upon near-infrared or thermal stimuli, allowing controlled payload diffusion and adaptive deformation at tissue interfaces [94–96, 135, 136]. Structural innovations, such as anisotropic alignment and double-network architectures, have further improved hydrogel resilience, achieving tensile strengths exceeding 6 MPa and stretchability beyond 1000% while maintaining responsive actuation fidelity [136, 137]. Through these design strategies, hydrogels are evolving from passive matrices into active, logic-capable materials that integrate sensing, actuation, and drug delivery within unified soft robotic platforms.

For biomedical integration, long-term in vivo stability remains a critical criterion determining translational feasibility. Traditional hydrogels often suffer from dehydration, swelling-induced delamination, or uncontrolled biodegradation in physiological media. To address these issues, organohydrogel and eutectogel systems incorporating antifreeze agents, dynamic covalent bonds, or hydrophobic networks have been engineered to preserve elasticity (up to 1500%) and conductivity across –30 to 60 °C [94–96, 136, 138]. Recent studies have begun to quantify degradation kinetics under simulated physiological conditions, showing tunable half-lives ranging from several days to over a month depending on polymer cross-link density and ionic composition. Such data underscore the importance of tailoring degradation pathways to match tissue regeneration and drug release timescales. Future integration of programmable synthesis and bioinspired hierarchical structuring is expected to yield hydrogels that not only maintain structural integrity and responsiveness in vivo but also synchronize therapeutic release with robotic actuation, advancing toward truly autonomous and life-mimetic biomedical soft robots [97, 139–143].

#### Liquid Crystal Elastomers and Their Composites

Liquid–crystal elastomers (LCEs) and their composites offer great potential for next-generation soft robotics owing to their programmable deformation, responsiveness, and multifunctionality. Inspired by skeletal muscle, hollow LCE (h-LCE) fibers with programmable outer shells and functional internal channels have enabled a range of applications, including water-triggered actuation, stiffness tuning with shape memory polymers (SMPs), and integration with liquid metals for electrically driven systems. These h-LCE fibers, which are fabricated at scales up to 3 m long and as small as 250 μm in diameter, support advanced designs for artificial muscles and soft robotic components [99]. At the microscale, LCEs are used to construct reconfigurable thermoresponsive metasurfaces via two-photon polymerization, enabling the creation of micrometer-scale origami structures. While wireless actuation remains a challenge, simulation-guided strategies are helping to optimize shape transformations and device geometries for soft microrobots and wearables [100]. A biomimetic spinning method inspired by spiders allows for scalable production of high-performance LCE microfibers with rapid strain, high stress, and long lifespans, supporting applications in smart textiles and humanoid robots [101].

**Fig. 4:**
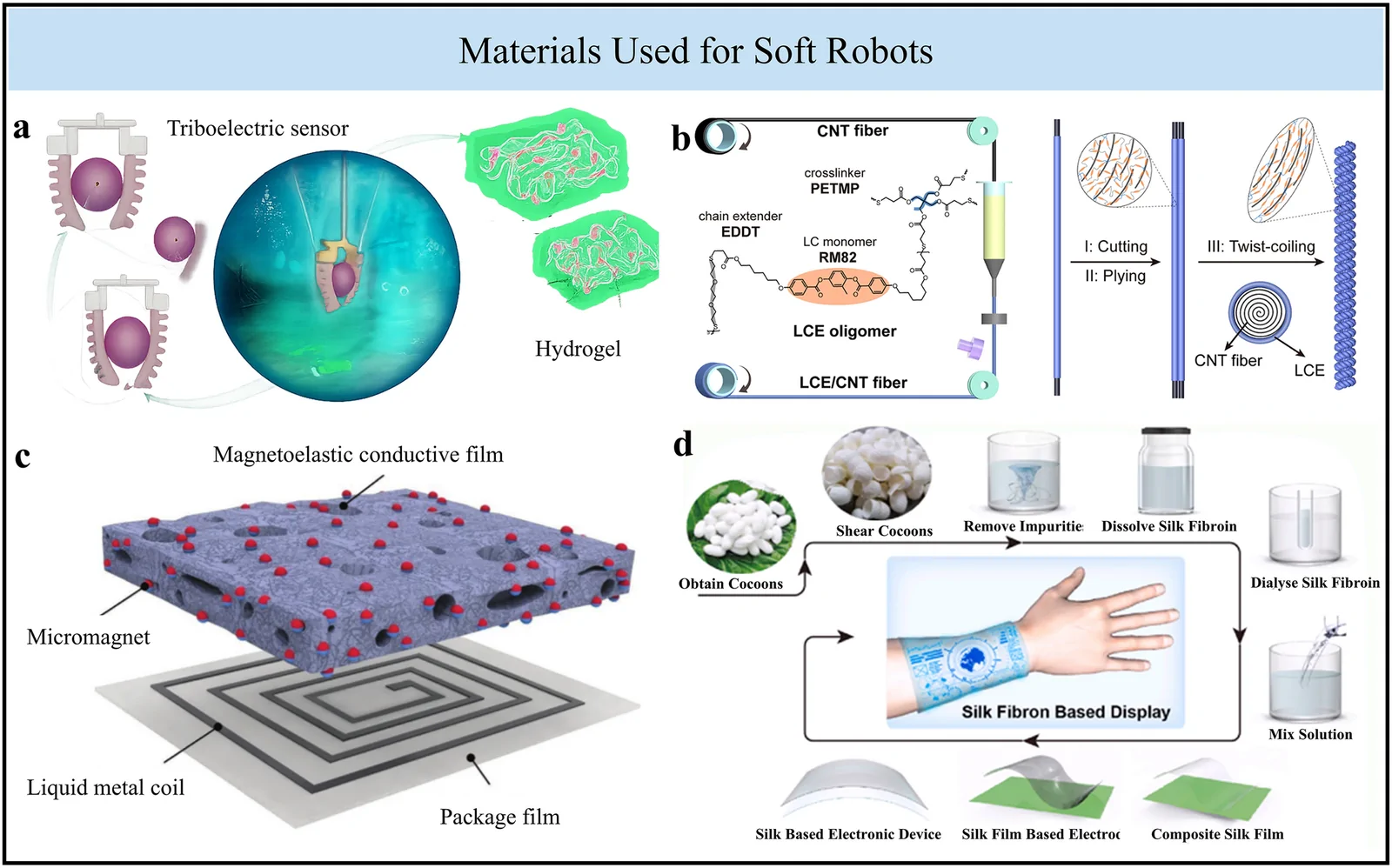
Four materials used in soft robots. **a** Schematic diagram of a soft robot gripper integrated with a strain triboelectric sensor based on the developed hydrogel and its formation mechanism [93]. Copyright 2023, Elsevier. **b** Schematic diagram of the continuous preparation of LCE/CNT composite fibers through LCE coating, fiber stranding and twisting [102]. Copyright 2023, American Chemical Society. **c** The dual-mode self-powered flexible sensor (BSFS) consists of two functional flexible films: a magnetoelastic conductive film and a liquid metal coil with a packaging film [144]. Copyright 2023, Wiley–VCH. **d** After undergoing processes such as washing, degumming, dissolution, dialysis, centrifugation, and doping, silk protein can be transformed into a flexible substrate material with excellent light transmittance and mechanical strength, making it suitable for use in various flexible wearable electronic devices [145]. Copyright 2023, Elsevier

In addition, a programmable flexible actuator (PFA) inspired by fig tree leaves was fabricated via 4D printing. Utilizing a composite of liquid metal, spider silk, and liquid crystal elastomers (LCEs), this actuator integrates sensing and selective actuation through precisely designed photothermal and mesophase architectures. The spatiotemporal rearrangement of microstructures enables remote, programmable deformation and signal transduction, advancing the development of adaptive human–machine interfaces [119]. Likewise, self-recovering coiled artificial muscle fibers were realized by coating LCE sheaths on elastic CNT fibers, achieving large reversible contractions and high response rates through Joule heating. These helically aligned LCE chains mimic natural muscle behavior for efficient motion tasks such as object manipulation and rapid actuation (Fig. 4b) [102]. At the microscale, 3D nanofabricated “pico springs” composed of acrylic elastomer photoresists provide tunable compliance and magnetic responsiveness, facilitating force-sensitive micromachines capable of delicate and biocompatible interactions with cellular systems [76].

#### Liquid–Metal Composite Materials

Liquid metals (LMs) and their composites offer unique advantages for next-generation soft robotics, including flowability, conductivity, and deformability [103, 146, 147]. However, conventional LMs face limitations such as high density, cost, and fragility [104]. To overcome these challenges, researchers have developed novel LM-based composites. For example, LM/CNT hydrogels exhibit high conductivity, stretchability and transparency for aquatic health monitoring [105]. LMPP hydrogels stabilized by PEDOT:PSS show increased strength and conductivity for motion tracking [106]. LM micronetwork films achieve conductivities of up to 2.37 × 10⁶ S m^−1^ under strain, which are ideal for wearable circuits [107]. PEN composite films made from PDMS, EGaIn, and NdFeB offer high moduli and tear strengths for soft robot actuation [108]. BTMN-SHFSS sensors inspired by human skin exhibit 100% electrical repair efficiency and broad-range sensitivity [104]. Similarly, spider web-inspired semi-LM electronic skin achieves high conductivity, stretchability, and breathability for long-term monitoring [148].

To reduce cost and density, LM fiber network composites with ultralightweight structures offer stable, strain-independent conductivity and cycling performance, enabling stretchable sensors [103]. LMS inks enable 3D-printed, self-encapsulated, flexible tactile sensors with excellent durability and human posture recognition [149]. LM-based triboelectric nanogenerators (LM-TENGs) generate output voltages of 22.29 V and 55.16 μW of power, along with impact protection for smart healthcare [150]. LMPSTs also demonstrate strong energy harvesting and force sensing capabilities [109]. LM-hydrogel sensors with wireless modules enable gesture and sign language recognition [103]. BSFS dual-mode sensors based on triboelectric and magnetoelastic effects can distinguish noncontact and tactile inputs and are integrated into robotic hands for high-precision object recognition via CNNs (Fig. 4c) [144].

Advanced fabrication methods have further broadened LM applications. Water-based ion chelation enables the continuous production of magnetic LM fibers with high mechanical and electrical stability for shielding and sensing. [151] Liquid metal solders with small-molecule modulation stretchability and toughness for deformable electronics [152]. Iron oxide-composited LMs enable magnetically driven microsoft robots via external fields [153]. Magnetized EGaIn@Fe materials allow 3D manipulation without electrolytes, supporting microfluidics and circuit repair [154]. Biomass-encapsulated LM/protein gels (GLMx) exhibit adhesion, toughness, and recoverability, serving as epidermal sensors for body motion monitoring [155]. Overall, LM research is advancing toward multifunctional, sustainable, and low-cost materials for flexible electronics, wearables, and robotics. Future work will focus on performance optimization and deployment in complex environments.

#### Protein-Based Smart Materials

With the rise of soft robotics and flexible electronics, protein-based materials have emerged as promising candidates due to their biocompatibility, biodegradability, and mechanical tunability. In water-responsive systems, 4D-printed zein gels enable programmable degradation and drug release by adjusting hydrogen bonding through ethanol–water mixtures, expanding 4D printing to include functional heterogeneity [110]. Spider silk-inspired sericin fibers display water-triggered shape memory and cyclic actuation with up to 18 MPa stress, making them suitable for actuators and smart textiles [111]. Sericin membranes offer multiple types of responsiveness, including thermal shrinkage and humidity response, with reprogrammable flipping and self-oscillation for applications such as biomimetic wings and soft grippers [112]. Silk protein films, with a surface-to-interior density gradient from asymmetric water diffusion, achieve excellent strength and moisture-driven origami and jumping behaviors [113]. These structural protein systems exploit hierarchical hydrogen-bond networks and water-mediated plasticity, providing versatile mechanical adaptability and tunable degradation for dynamic environments.

For self-powered and biointegrated sensing, protein-based triboelectric and piezoelectric devices leverage structural hierarchy to improve charge generation, mechanical resilience, and biocompatibility. Keratin-based S-TENGs incorporating CaCl₂ and Ecoflex exhibit enhanced voltage output, stability, and stretchability, enabling precise gesture and shape recognition for biomedical and environmental applications [110]. The hexagonal keratin structure of snake skin provides porosity and pressure sensitivity for the development of sustainable piezoelectric and triboelectric devices [114]. Silk/PVBVA nanofiber-based TENGs deliver high power for joint monitoring with waterproof, recyclable, wearable properties ideal for IoT systems [115]. Silk protein elastomers with PDA-intercalated clay offer superior adhesion and flexibility for epidermal sensing of vibration and voice signals [156]. Silk electrodes with improved stability via mesoscale doping support flexible display integration (Fig. 4d) [145]. Advanced silk/MXene composite films achieve a sensitivity of 17.1 kPa⁻^1^ over the 3.3 MPa range and are applied in motion detection and human‒machine dialog systems [157]. A thiol-ene click chemistry-based BSA hydrogel enables freeze-resistant, biocompatible, conductive sensing for gesture-based robotic control [158, 159]. For biomedical applications such as implantable microneedle robots for gastrointestinal drug delivery, long-term in vivo stability is crucial. Existing studies report that silk fibroin microneedles retain mechanical integrity for approximately 12–48 h in simulated gastric fluid and several days in intestinal environments before enzymatic degradation, depending on β-sheet crystallinity and cross-linking density. Such degradation kinetics can be modulated through methanol annealing, ion doping, or silk–polymer hybridization, ensuring controlled dissolution and mechanical endurance while minimizing fibrotic encapsulation or immune responses. In integrated bioelectronic systems, amyloid fibrils self-assembled at the air‒water interface can be combined with CNTs or Fe₃O₄ for magnetically responsive soft robotic swimmers and sensors [160]. Wool keratin enables bioelectronic platforms with stable, conductive CNT dispersions for ECG electrodes and flexible circuits [161]. Dry-spun silk-based ion‒electronic fibers (SSIFs) demonstrate rapid electromechanical response and, when combined with triboelectric fibers and machine learning, enable material classification for human‒machine interfaces [162]. Devices using FWCNTs and silk-assisted transfer technology exploit intershell sliding to provide a stable electrical response at next-generation interfaces [163]. Finally, Ti₃C₂Tx-Ag@silk nanofiber composites with heterogeneous conductive networks enable fast-heating paper devices, high-efficiency EMI shielding, and high-sensitivity capacitive pressure sensing for gesture recognition and wireless control applications [164].

## Intelligent Drive and Energy Management

### Flexible Drive and Motion Control

**Fig. 5:**
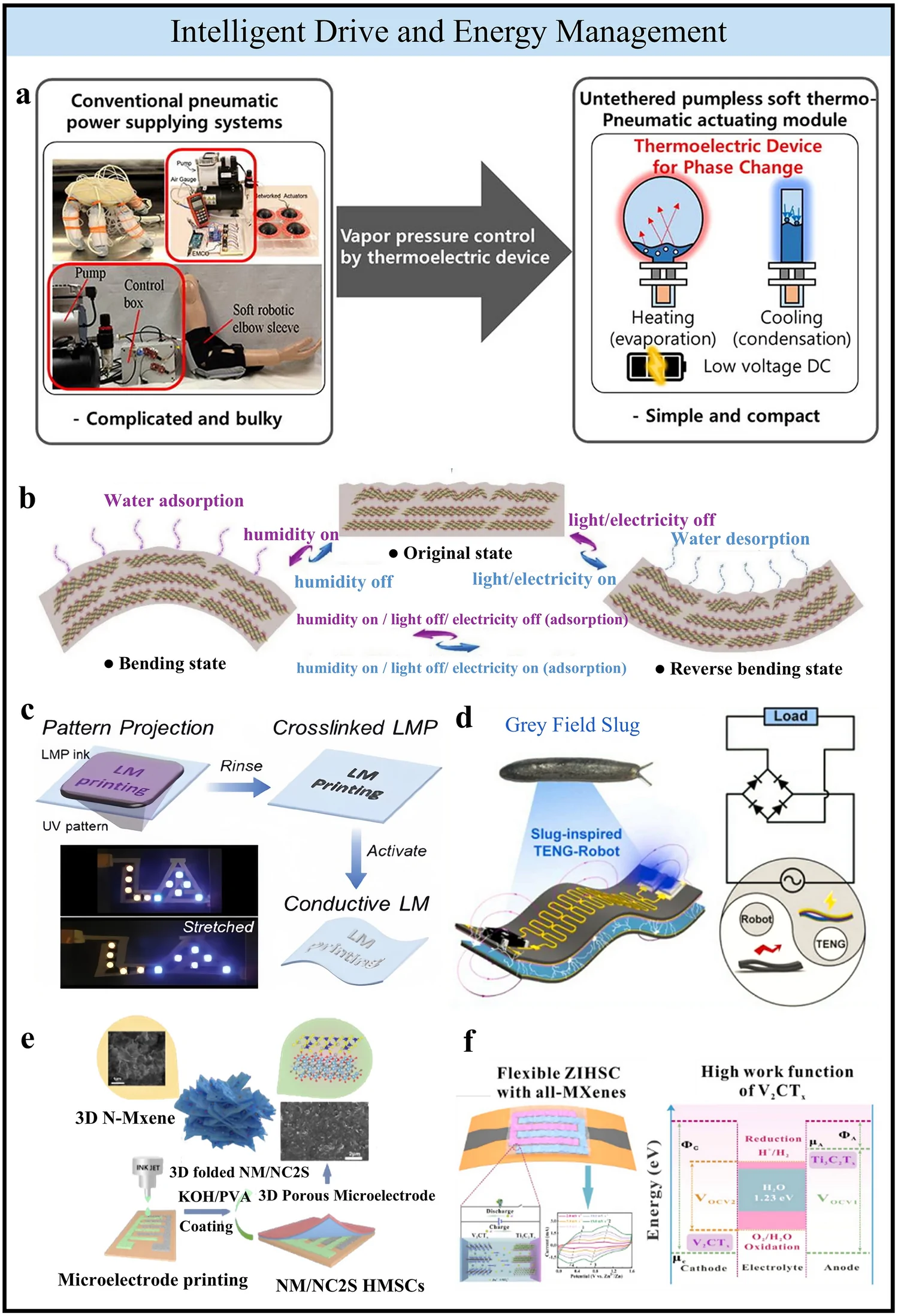
Intelligent drive and energy management. **a** Function of the Thermally Controlled Pneumatic Structure [165]. Copyright 2023, Elsevier. **b** Formation mechanism of biomimetic motion patterns in MXenes and other multiresponsive materials [116]. Copyright 2024, Wiley–VCH. **c** The use of liquid metal and high-resolution lithography enables soft actuators to achieve increased integration and mechanical compatibility [166]. Copyright 2024, Wiley–VCH. **d** The use of liquid metal and high-resolution lithography enables soft actuators to achieve increased integration and mechanical compatibility [167]. Copyright 2022, Elsevier. **e** Hybrid structures based on MXenes can increase energy storage efficiency and structural stability [117]. Copyright 2024, Elsevier. **f** Role of the porous network and three-dimensional electrode introduction [118]. Copyright 2023, Elsevier

Flexible drive technology is the foundation for soft robots to achieve biomimetic motion and complex shape reconstruction, with its core lying in the development of functional materials and structural systems capable of responding to various external stimuli. In recent years, various actuation strategies, such as thermally induced phase change, electrically induced deformation, light-responsive, and magnetically controlled materials, have been developed, expanding the motion freedom and response scenarios of soft robots. Among these, thermally controlled pneumatic structures utilize thermoelectric elements to achieve rapid expansion and contraction of gas within chambers, enabling fully soft-driven systems without external air pumps [165] (Fig. 5a). Multiple responsive materials, such as MXenes, can generate direct deformation under humidity, temperature, and electric field stimuli by constructing asymmetric nanofluidic channels, thereby achieving more complex and biomimetic motion patterns [116] (Fig. 5b). Additionally, by leveraging liquid metals and high-resolution lithography techniques, the construction process of drive circuits is increasingly moving toward modularization and miniaturization, enabling soft actuators to achieve higher integration and mechanical compatibility [166] (Fig. 5c). These multiphysics field-coupled drive mechanisms provide a solid foundation for developing autonomous, programmable soft robots.

Recent studies have demonstrated that porous microneedle (MN) architectures can effectively bridge the functional gap between traditional actuator materials and biological tissues, offering both mechanical adaptability and multi-stimuli responsiveness. By integrating interconnected microchannels and gradient porosity within the MN array, internal stress distribution can be precisely modulated under external stimuli such as heat, light, or electrochemical potential. This enables anisotropic expansion, rapid solvent diffusion, and localized drug release, all of which can be harnessed to generate controlled deformation or locomotion in soft robots. For instance, a hygroresponsive MN array composed of silk fibroin and graphene oxide demonstrates reversible bending and twisting motions driven by differential water absorption across the needle height, mimicking the contraction of muscle fibers while simultaneously realizing on-demand drug delivery to adjacent tissues. Similarly, photothermal porous MN actuators based on polydopamine-coated hydrogels have been shown to achieve millimeter-scale displacement within seconds under near-infrared (NIR) irradiation, where the hierarchical pore structure not only accelerates heat transfer and water evaporation but also improves actuation frequency and durability during cyclic operation.

Furthermore, the coupling of porosity and electrical conductivity has opened new opportunities for integrating MN-based actuators with energy harvesting or sensory modules, enabling self-regulated motion control. In one representative system, liquid–metal-filled porous MNs exhibited dynamic stiffness tuning and real-time feedback regulation: As the LM channels deformed under stress, their resistive network simultaneously generated sensing signals for closed-loop control, thereby achieving synchronized actuation and sensing within a single soft module. Such designs directly address limitations in power density, response precision, and functional lifetime that have long constrained soft robotic systems. The incorporation of porous MN arrays as both mechanical transducers and functional interfaces represents a significant step toward autonomous, adaptive, and self-sustaining soft robots capable of performing complex biomedical or environmental tasks.

### Microneedle Array-Based Local Actuation and Energy Harvesting

Recent advances in microneedle (MN) array systems have revealed their potential not only as passive transdermal interfaces but also as active platforms for localized actuation and on‐site energy harvesting within soft robotic architectures. The integration of self‐powered biochemical and triboelectric systems into MN matrices has enabled the coupling of therapeutic drug delivery with dynamic mechanical or electrical output, forming a closed‐loop regulation paradigm. For instance, Zhang et al*.* developed a self‐powered enzyme‐linked MN patch composed of anodic and cathodic arrays encapsulating glucose oxidase and horseradish peroxidase within ZIF-8 nanoparticles [168]. The enzymatic cascade simultaneously consumes excess glucose in diabetic wounds and generates stable microcurrents, achieving synergistic regulation of biochemical microenvironments and electrostimulation. Such bioelectric feedback not only accelerates angiogenesis and collagen remodeling but also establishes a model for autonomous energy utilization in soft therapeutic robots. These MN patches exemplify how localized enzymatic reactions can transform endogenous biochemical energy into electrical stimuli, realizing wound healing or regeneration without external power inputs.

Building upon this concept, Wang et al*.* reported a wearable self‐powered MN patch integrating a flexible triboelectric nanogenerator (F-TENG) for deep‐seated melanoma therapy [169]. The mechanical deformation of the host tissue or the wearer’s motion is converted into electrical energy, which subsequently drives iontophoretic enhancement and controlled drug release from pH-responsive nanoparticles embedded within water‐soluble MNs. This design demonstrates synchronous coupling between motion‐derived energy and therapeutic actuation—each mechanical movement of the body translates into proportional stimulation and drug transport. Similarly, Chen et al*.* introduced a self‐powered controllable MN system capable of achieving rapid antihypertensive drug release through a pressure-triggered bioelectric circuit [170]. Together, these approaches highlight the dual roles of MN arrays as localized actuators and energy harvesters: one that bridges chemical energy conversion, microcurrent regulation, and dynamic drug delivery. In the context of soft robotics, such hybrid MN platforms offer an elegant route toward distributed, wire-free control—enabling autonomous, site‐specific actuation and energy feedback for self‐regulated therapeutic or sensing tasks.

### Self-Powered Systems

Soft systems impose nontraditional requirements on power supplies, demanding energy units that are not only flexible but also adaptable to complex and dynamic environments. Self-powered mechanisms—including triboelectric, biofuel, and thermoelectric systems—offer a promising route to meet these needs by generating energy in situ, eliminating reliance on bulky external power sources. For instance, sweat-driven biofuel cells exploit the reducing agents present in human perspiration to produce voltages sufficient to actuate electroactive polymers, enabling self-induced bending and motion output [171]. Triboelectric nanogenerators (TENGs) operate through contact-separation-induced charge accumulation, providing a dynamic power supply that harvests energy during motion [167] (Fig. 5d). Quantitatively, typical TENGs generate power densities on the order of 1–100 μW cm^−2^ under standard laboratory motion conditions, which, while sufficient for low-power sensors or small actuators, remains significantly lower than commercial flexible batteries (in the mW/cm^2^ range). Thermoelectric systems leveraging solar absorption and radiative cooling can produce continuous voltage outputs during daytime or in high-temperature environments, supplying stable energy for wearable or soft robotic devices [172].

Despite their potential, these self-powered systems face critical practical limitations that must be addressed to enable real-world deployment [173]. TENGs, for example, often exhibit performance degradation under repeated robotic motion due to surface wear, charge leakage, and environmental sensitivity, particularly to humidity and mechanical contamination. Their low output poses a bottleneck for high-power actuators or sustained operations, highlighting the need for design optimization. Recent advances in material and structural engineering offer potential solutions: liquid–metal-infused TENGs (LM-TENGs) and surface microstructuring have been demonstrated to enhance mechanical robustness, maintain charge density under repeated deformation, and reduce sensitivity to moisture [174]. Similarly, sweat-driven biofuel cells face limitations in energy density and temporal stability, which can be partially mitigated through nanostructured electrodes or enzyme immobilization strategies that prolong catalytic activity. By linking these material and structural innovations directly to system-level performance, soft robotics can transition from proof-of-concept demonstrations toward reliable, self-sufficient operation in complex, real-world environments [175].

### Flexible Energy Storage Devices

Flexible energy storage technology provides a critical foundation for the long-term independent operation of soft robots. An ideal energy storage unit should possess high energy density, excellent cycling performance, and the ability to maintain stable electrochemical performance under deformation conditions. Current research focuses on the material design and structural optimization of novel devices such as microsupercapacitors and flexible zinc-ion batteries. Hybrid structures based on MXenes can achieve a reasonable distribution of interfacial electron density while ensuring conductivity, thereby increasing energy storage efficiency and structural stability [117] (Fig. 5e). The introduction of porous networks and three-dimensional electrodes further enhances the ion diffusion and electron transport pathways, enabling the devices to maintain excellent performance under high-frequency charging and discharging as well as multiple mechanical bending cycles [118] (Fig. 5f). The continuous optimization of flexible energy storage components provides a stable power supply for integrated power supply, electronic control, and sensing modules in soft robotics systems, which is particularly suitable for operation under dynamic loads or in wearable scenarios.

## New Developments in the Integration of Soft Robotics and Artificial Intelligence

### Artificial Intelligence-Enhanced Soft Sensing and Interaction

**Fig. 6:**
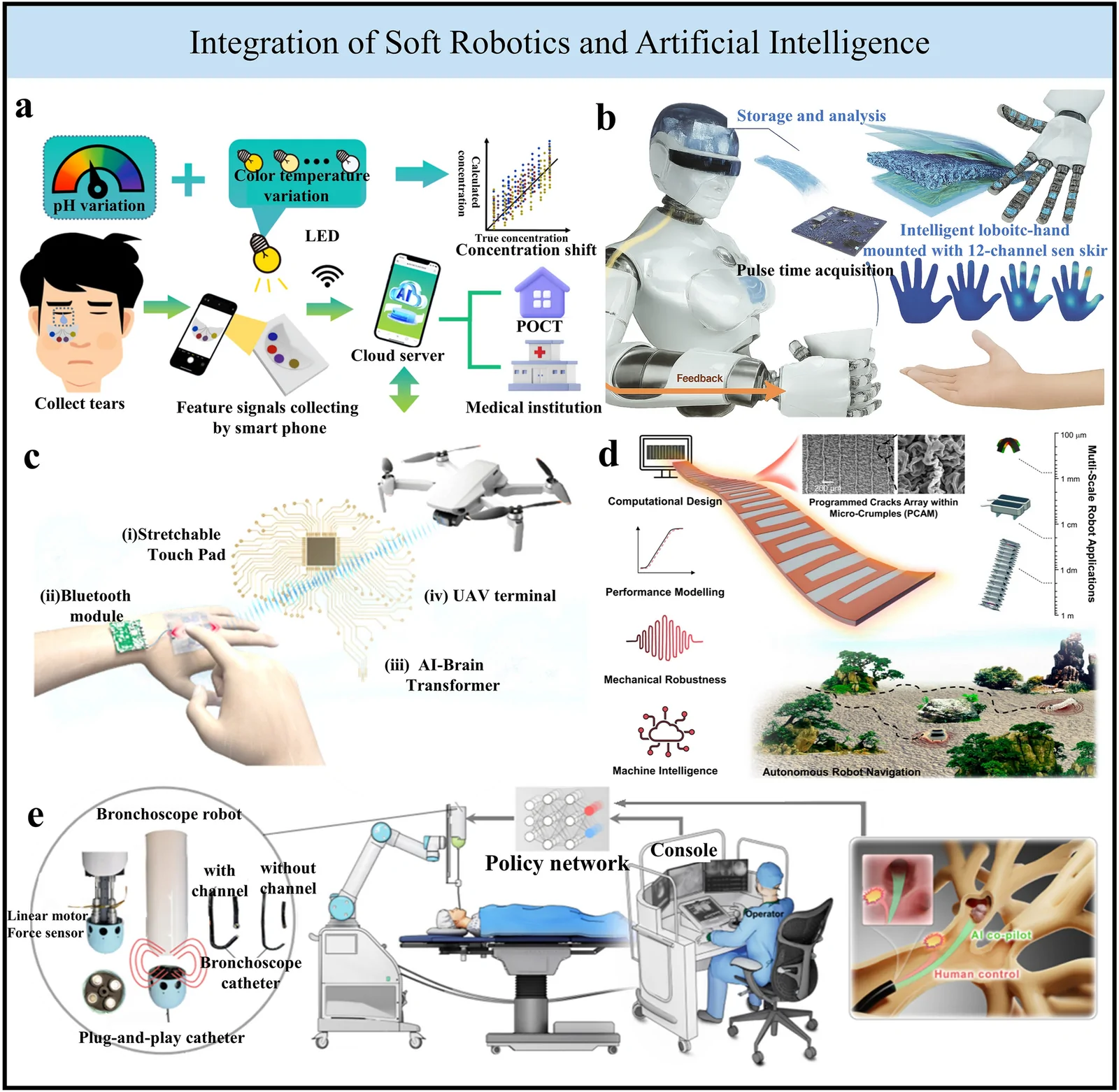
**a** Basic concepts of colorimetric sensing of key biomarkers in human tears via an AI-assisted microfluidic colorimetric wearable sensor system [176]. Copyright 2024, Springer Nature. **b** Working mechanism of the wearable Morse code-to-speech translation system [177] Copyright 2023, Science China Press. **c** Schematic diagram of drone control in extreme environments via ultraflexible triboelectric touchpads [178]. Copyright 2023, American Association for the Advancement of Science. **d** Improving the computational design process of ultrrobust strain sensors for autonomous soft robot self-awareness [180]. Copyright 2024, Springer Nature. **e** Schematic diagram of a remotely controlled AI-assisted bronchoscope robot performing lung examinations in a clinical setting [181]. Copyright 2024, Springer Nature

A key advancement in the field of soft robotics lies in the significant improvement of its perception and interaction capabilities, which is largely attributed to the deep integration of artificial intelligence (AI) technology. Traditional soft sensors face challenges in data collection, interpretation, and sharing, which limits their potential in practical applications. To overcome these limitations, researchers have developed various AI-assisted soft sensing systems aimed at achieving more precise, multimodal, and real-time environmental perception and human‒robot interaction [176] (Fig. 6a). For example, in the field of biomedical monitoring, an AI-assisted microfluidic colorimetric wearable sensor system has been proposed for rapid, noninvasive, and synchronous detection of key biomarkers in human tears. Similarly, in robotics perception, researchers have developed an intelligent soft robotics system based on bimodal self-powered flexible sensors (BSFS) that can perceive, describe, and classify objects, achieving up to 97% accuracy through convolutional neural networks (CNNs) [144]. Additionally, AI plays a crucial role in enhancing the perceptual capabilities of electronic skin (e-skin), enabling a wearable Morse code-to-speech translation system with high recognition accuracy through deep learning algorithms [177] (Fig. 6b) as well as an integrated intelligent tactile system (IITS) integrated into humanoid robots to achieve flexible grasping [178] (Fig. 6c). Zero-bias biomimetic fingertip electronic skin (E-skin) combines machine learning and feature fusion to comprehensively perceive objects, distinguish surface roughness and hardness, and accurately identify objects at different temperatures [179].

AI is also used to enhance the environmental adaptability and complex task execution capabilities of soft robots. For example, a soft robot perception system based on ultrasonic automatic positioning and multimodal perception intelligence integrates ultrasonic sensors and flexible triboelectric sensors to detect object shapes and distances, enabling the robotic arm to locate and execute grasping tasks precisely, with nearly 100% object recognition accuracy achieved through deep learning analysis [182]. In extreme environments, an ultrastretchable triboelectric touchpad integrates liquid metal and high-performance hydrogel-based triboelectric sensor arrays combined with transformer-assisted gesture recognition technology to achieve high-precision real-time gesture recognition for drone flight direction control [183]. These studies collectively demonstrate the immense potential of artificial intelligence in enhancing soft sensing and interaction capabilities, enabling soft robots to perceive their own state and surrounding environment more precisely while achieving smarter, more adaptive behaviors [184, 185].

Beyond direct perception, AI also plays a role at more abstract levels of perception and interaction. Machine learning-assisted electronic skin can be integrated into soft robots to reconstruct their shape during movement (proprioception) and identify various terrains (exteroception), suggesting more advanced autonomous robots [186]. AI-enhanced hardware supports the application of multimodal electronic skin to rescue robots, enabling them to possess robust environmental perception capabilities, such as precisely distinguishing objects and identifying human limbs through grasping, and even real-time detection of toxic gases. In the field of medical diagnosis, wireless multimodal wearable systems combined with machine learning algorithms can automatically and accurately assess swallowing behavior and diagnose silent aspiration, offering a promising noninvasive alternative for swallowing disorder healthcare and rehabilitation therapy [187]. Additionally, passive addressable robot metamorphic surfaces (PARMS) achieve real-time, high-precision forward and inverse control of matrix-arranged ion actuators through training that combines machine learning and finite element simulation, enabling them to dynamically deform into any predefined surface on demand. This is significant for wearable devices, haptic feedback, and augmented/virtual reality [188]. These advancements are pushing soft robotics to the forefront of revolutionary changes across multiple fields, including healthcare, rescue operations, and human‒machine interactions.

### Application of Artificial Intelligence in Soft Robot Design and Control

The application of artificial intelligence in the design and control of soft robots has greatly promoted the performance optimization and autonomous capabilities of soft robots. In terms of design, computational intelligence methods are widely used to create soft robots with specific functions and morphologies. In terms of mobility, researchers have designed dexterous electrically driven soft robots with reconfigurable chiral lattice legs, achieving precise motion control [189]. In terms of autonomous behavior, unconstrained soft microrobots have demonstrated the potential to perform advanced tasks in complex environments by integrating adaptive logic gates [190]. Additionally, through action inheritance mechanisms, soft robots can rapidly evolve, enabling them to efficiently learn and adapt when performing new tasks [191]. For the perception and autonomy of soft robots [180] (Fig. 6d), computational design is used to develop ultrarobust strain sensors, providing reliable internal perception data for soft robots.

At the control level, artificial intelligence enables soft robots to handle complex dynamic environments by providing real-time feedback and adaptive algorithms. An example is a multifunctional soft robot shape display that combines high-speed drive [192], sensing, and control functions, enabling it to respond quickly and change shape to perform specific tasks. Furthermore, considering the inherent inertial dynamics of soft robots, researchers have proposed control methods tailored to their inertial dynamics, ensuring stability and precision during high-speed or complex movements [193]. These studies demonstrate how artificial intelligence comprehensively enhances the functionality and autonomy of soft robots, from conceptual design to practical implementation [194].

### AI Applications of Soft Robots in Specific Fields

Soft robots are increasingly being applied in specific fields because artificial intelligence technology endows them with greater adaptability and decision-making capabilities [195–197]. Soft robots have unique advantages in the exploration of complex or hazardous environments. An amphibious microsoft jumping robot can perform jumping movements in different media and achieve on-demand manipulation in mid-air, significantly expanding its application scope in reconnaissance or exploration tasks [198]. In the medical field, the integration of artificial intelligence and soft robots has led to revolutionary progress [181] (Fig. 6e). AI-assisted bronchoscope robots can assist doctors in performing precise lung examinations and treatments, increasing the safety and efficiency of medical procedures. Additionally, the integration of soft wearable flexible bioelectronic devices with ankle‒foot exoskeletons enable the estimation of metabolic costs and physical fatigue levels, providing personalized solutions for rehabilitation therapy and movement assistance.

Soft robots have also been endowed with more advanced intelligent behaviors to cope with unstructured environments and biological systems. For example, a physically intelligent autonomous soft robot maze escaper demonstrates the ability to autonomously solve complex path problems without external control [199]. In drug delivery, soft robots can mediate autonomous adaptation to fibrotic capsule formation, thereby improving drug delivery efficacy and addressing the challenge of drug absorption within the body [200]. In cardiovascular research, soft robots are used to construct patient-specific fluid dynamics models of aortic valve stenosis and ventricular remodeling [182], providing new tools for personalized diagnosis and treatment. Finally, self-folding soft robot chains demonstrate their reconfigurable shapes and functions [201], enabling them to adapt to various task requirements, which is significant for the development of morphologically variable tools and multifunctional systems. These cases highlight the critical role of artificial intelligence in driving breakthroughs in soft robotics across diverse fields, such as medicine, exploration, and autonomous systems.

## Intelligent Sensing and Biointeraction

### Multimodal Perception and Human‒Computer Interaction

**Fig. 7:**
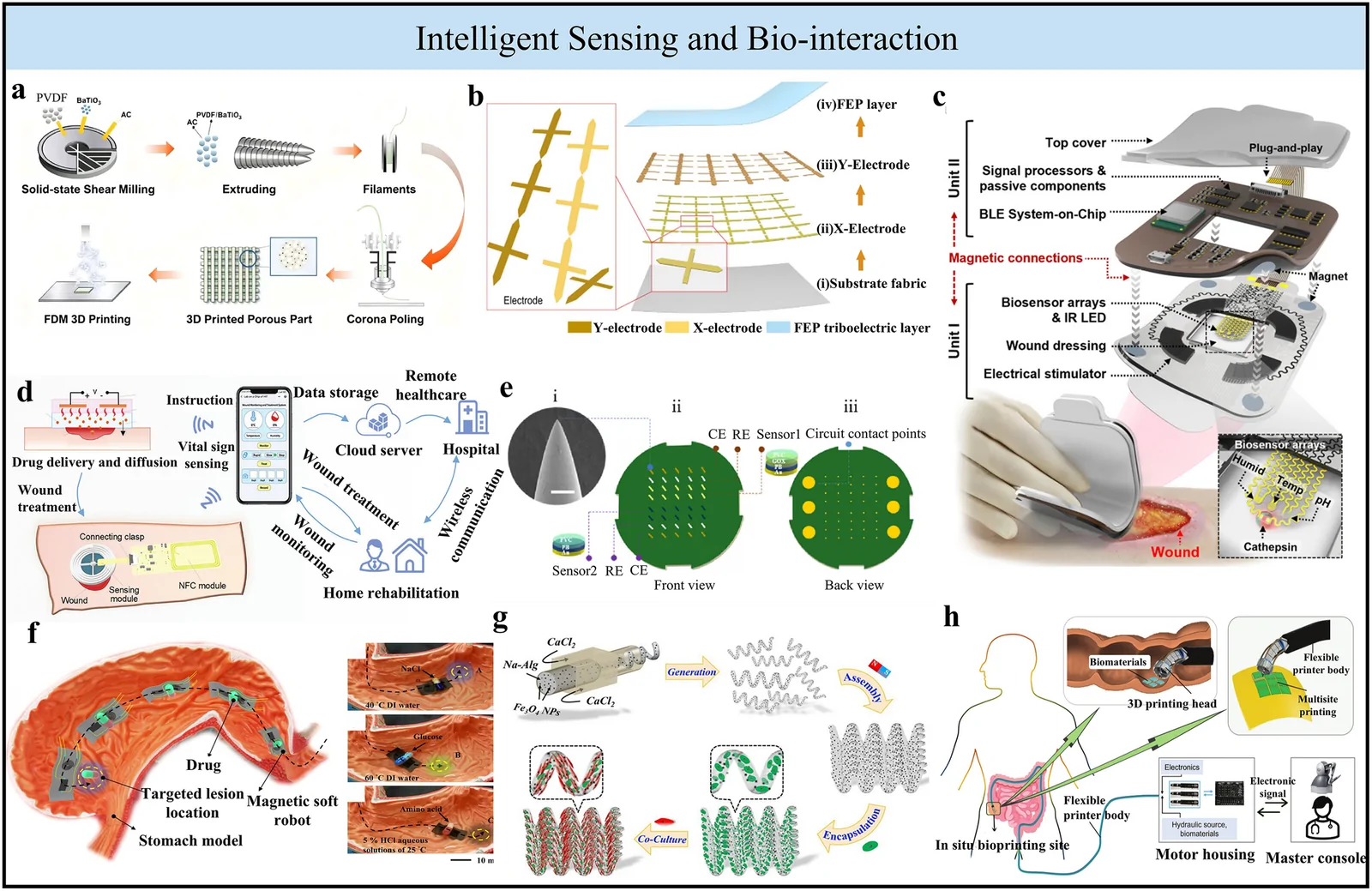
**a** 3D printing additive manufacturing PVDF/BaTiO3 MMH-PENG sensor process diagram [202] Copyright 2024, Elsevier. **b** Branch structure of the bionic DES electrode unit [203] Copyright 2024, Springer Nature. **c** Schematic diagram of the layered unit of the electronic/optical wound monitoring/healing system. Unit I contains monitoring components for temperature, humidity, pH, and inflammatory markers [205]. Copyright 2022, Elsevier. **d** In the IWD wound treatment and information collection, the smartphone mini-program receives and stores data and feeds back the test results as a controllable closed-loop treatment system workflow diagram with electric heat and light stimulation [206]. Copyright 2023, Wiley-Blackwell. **e** Microneedle arrays were prepared using 160 μm diameter acupuncture needles (i). Scale bar, 30 μm. Glucose sensors and differential sensors were prepared on a microneedle array (ii). The back of the microneedle array has electrode contacts connected to a signal acquisition system (iii) [207]. Copyright 2024, Elsevier. **f** Schematic diagram of magnetically controlled targeted drug delivery in gastric models with different liquid environments using magnetic soft robots [208]. Copyright 2023, Elsevier. **g** Spiral micromotor structure, magnetic control assembly, and cell nanoparticle encapsulation [209] Copyright 2022, Elsevier. **h** In vivo 3D printer (F3DB) schematic diagram of in situ printing on the surface of in vivo tissue [210]. Copyright 2023, Wiley–VCH

In the process of soft robotics evolving toward intelligence, enhancing perception capabilities is a critical step in achieving environmental interaction, behavioral feedback, and autonomous decision-making. Flexible sensors based on triboelectric, piezoelectric, thermoelectric, and resistive strain principles can detect various physical signals from the external environment with high sensitivity and possess excellent mechanical compliance and multifield response capabilities. Through structural design optimization, such as hierarchically structured design at the macro- and microscales, buckling folding configurations, and 3D printing manufacturing processes, sensor arrays can simultaneously perform real-time monitoring of multiple inputs, such as bending, stretching, and pressure [202, 203] (Fig. 7a, b). Additionally, triboelectric sensor arrays can achieve signal channel compression through digital modeling and cross-array design, reducing system complexity while enhancing spatial resolution and response efficiency [204]. These high-throughput, multimodal sensing solutions are widely applied in human‒machine interface systems, virtual reality devices, and wearable identification systems, further expanding the application boundaries of soft robots in the service and assistive domains.

Despite these advances, soft robotic systems still face significant challenges in achieving stable and adaptive biointeraction. The primary difficulties lie in maintaining long-term biocompatibility and adhesion at the dynamic tissue interface, minimizing motion artifacts and hysteresis during continuous deformation, and ensuring signal fidelity in wet or ion-rich physiological environments. For instance, biofluid infiltration and protein adsorption can deteriorate triboelectric output stability, while enzymatic degradation or fibrotic encapsulation impairs the sensitivity of implantable sensors over time. Furthermore, the mismatch between the mechanical modulus of soft sensors and native tissues may lead to stress concentration or immune activation during chronic operation.

To address these limitations, structural and material innovations are being actively explored. The introduction of macro–microhierarchical architectures can effectively decouple strain transfer and suppress mechanical fatigue, as demonstrated by Han et al. [202] where hierarchical piezoelectric nanogenerators achieved enhanced charge retention and stable output under repeated bending through multiscale interlocking geometries. Similarly, triboelectric gloves integrating pneumatic actuation and sensor fusion, reported by Wang et al. [204], exemplify the potential of integrating active haptic feedback and multimodal perception in a unified soft interface, thus enhancing tactile communication and bidirectional interaction. From a biomedical perspective, strategies such as surface functionalization with zwitterionic or hydrogel layers can reduce immune responses and biofouling, while ionically conductive hydrogels or protein–polymer hybrid networks provide long-term stability and adaptive mechanical matching in vivo. Additionally, closed-loop multimodal fusion systems that combine self-powered sensing, machine learning algorithms, and soft actuation enable real-time physiological feedback and intelligent motion adjustment. Collectively, these approaches pave the way toward biointeractive soft robots capable of continuous sensing, adaptive response, and long-term operation within complex physiological environments.

### Medical Perception and Treatment Systems

The development of soft electronic devices in medical applications is characterized by the bidirectional integration of monitoring and therapeutic functions. By integrating multiple physiological sensing modules on flexible substrates, these systems can acquire real-time information such as temperature, humidity, pH, and inflammatory markers in the wound microenvironment, enabling dynamic assessment of the wound healing status [205] (Fig. 7c). Additionally, integrated electrothermal and optical stimulation modules can respond on demand based on the monitoring results, establishing a closed-loop control treatment process [206] (Fig. 7d). Owing to their minimally invasive and continuous sampling characteristics, microneedle array structures are commonly used for detecting biomarkers such as glucose and lactate in biofluids. Their regional addressability and differential amplification technologies further enhance detection accuracy and signal stability [207] (Fig. 7e). Collectively, these technologies are transforming medical systems from passive monitoring platforms to active, adaptive, and responsive therapeutic systems, particularly suitable for chronic disease management and personalized treatment needs, providing a demonstrative pathway for the evolution of intelligent soft medical devices.

However, the transition of these systems from laboratory prototypes to clinical applications requires careful consideration of biosafety and long-term biocompatibility. Despite their excellent sensing performance, soft electronics and microneedle-based robotic systems inevitably face biological risks associated with chronic implantation or prolonged skin contact, including enzymatic degradation, fibrotic encapsulation, and immune rejection. For instance, in vivo degradation of polymeric hydrogels and protein-based scaffolds can lead to pH fluctuations and byproduct accumulation, which alter local tissue homeostasis. Additionally, soft electrodes or microneedles inserted into dynamic tissues may cause micromotion-induced inflammation and fibrous tissue deposition, compromising signal quality and drug release efficiency. Recent studies have proposed material-level solutions to mitigate these risks. Duan et al. developed a water-modulated biomimetic “Hygel” electronic skin exhibiting weak acidity, antibacterial activity, and excellent biodegradability, achieving long-term skin compatibility through balanced hydration and interfacial buffering [53]. Similarly, Beatty et al. introduced a FibroSensing Dynamic Soft Reservoir (FSDSR) that senses and adapts to fibrotic capsule formation in vivo by modulating actuation frequency, effectively preserving drug delivery efficiency over extended periods [200]. These strategies underscore that controlling biochemical reactivity and fibrotic dynamics at the device–tissue interface is fundamental for ensuring biosafety in future soft robotic therapeutics.

From a broader perspective, current challenges in biointeraction involve mechanical mismatch between devices and living tissues, limited long-term electrical or chemical stability, and insufficient understanding of immune–mechanical coupling during chronic operation. The development of deployable, adaptive soft robotic systems capable of autonomous reconfiguration offers a promising pathway forward. Song et al. reported a pressure-driven deployable electrocorticography system using soft robotic eversion to achieve minimally invasive brain surface mapping, reducing cortical damage and enhancing recording fidelity [211]. In parallel, He et al. demonstrated layer-by-layer self-assembled thermoelectric fabrics with high breathability and stability after 2000 bending cycles and 600 washing cycles, highlighting that durable, breathable architectures can sustain physiological integration under long-term use [212]. Moving forward, improving interfacial adhesion, self-healing capacity, and immune invisibility of soft materials, combined with real-time feedback control and adaptive actuation, will be key to expanding their applicability in biomedical scenarios. The ultimate goal is to develop soft robotic systems that not only sense and respond, but also learn and adapt—achieving dynamic equilibrium between artificial devices and living tissues for personalized, sustainable, and intelligent healthcare.

### Tissue Engineering and In Vivo Manipulation

Tissue engineering and in vivo micromanipulation impose extremely high demands on the flexibility, size, controllability, and biocompatibility of devices. Spiral micromotor structures fabricated via microfluidic technology can encapsulate cells and functional nanoparticles, and their movement trajectories and assembly states can be regulated via magnetic control, demonstrating the potential for constructing complex three-dimensional tissue architectures [208, 209] (Fig. 7f, g). In vivo 3D printing devices coordinate high-degree-of-freedom flexible robotic arms with micronozzles to perform multilayer, multimaterial in situ construction on tissue surfaces, effectively addressing the structural deformation and contamination issues faced by traditional in vitro fabrication approaches [210] (Fig. 7h). These highly integrated, miniaturized soft systems hold great promise as key technological platforms for regenerative medicine, minimally invasive surgery, and precision disease intervention.

Despite these breakthroughs, the clinical translation of such microrobotic and biofabrication technologies remains subject to stringent regulatory and biosafety requirements. Current international standards—such as ISO 10993 for biological evaluation of medical devices, FDA 510(k) premarket notification for class II devices, and EU MDR 2017/745—mandate systematic assessments of cytotoxicity, local tissue reactivity, degradation kinetics, and sterilization validation. For soft microrobots and in vivo printing platforms, additional regulatory complexity arises from their hybrid material composition and functional actuation. Devices combining biodegradable polymers, hydrogels, and magnetic nanoparticles must demonstrate both mechanical reliability and predictable degradation in physiological fluids, while ensuring that magnetic or electroactive components do not trigger inflammatory cascades or interfere with local electrophysiological signals. Establishing unified performance benchmarks,such as minimal fibrotic encapsulation thickness (< 100 μm over 28 days) and controlled degradation half-lives (7–21 days for hydrogel-based scaffolds),will be critical for achieving regulatory approval and clinical adoption.

At the same time, biointeraction and long-term adaptability remain central challenges for soft robotic systems in tissue engineering. Mechanical mismatch between synthetic structures and native extracellular matrices can lead to chronic irritation, while biofouling, immune activation, and mechanical fatigue degrade actuation performance over time. Strategies to overcome these obstacles are increasingly focused on material-level biointegration and systemic intelligence. For example, Yue et al. designed a triboelectric nanogenerator–based tissue battery that converts mechanical joint energy into electrical stimuli, enabling continuous chondrocyte activation and real-time monitoring of cartilage repair [213]. Chen et al. further demonstrated an untethered artificial muscle driven by a sweat-based energy generator, forming a closed-loop system capable of powering self-regulated actuation for muscle rehabilitation [171]. In parallel, Zhai et al. proposed a monolithic 3D printing strategy to fabricate pneumatic soft robotic devices with embedded fluidic control circuits, allowing autonomous gripping and adaptive feedback without post-assembly [214]. Meanwhile, high-throughput magneto-origami fabrication has enabled scalable production of magnetically reconfigurable micromachines, pushing the field toward clinically viable mass customization [215].

Going forward, advancing the biomedical applicability of soft robotic systems will depend on the synergistic integration of biocompatible material chemistry, intelligent control algorithms, and regulatory compliance design. Combining in vivo–stable, self-healing materials with real-time feedback from embedded biosensors could enable autonomous adaptation to fibrotic encapsulation or biochemical stress. Furthermore, early alignment with clinical regulatory frameworks and ethical guidelines will accelerate safe translation from benchtop demonstrations to bedside applications. These efforts will collectively transform current soft microrobots from transient experimental platforms into clinically deployable agents for personalized, minimally invasive, and regenerative therapies.

## Conclusions and Perspective

Soft robots, constructed from responsive materials and bioinspired architectures, continue to redefine the interface between artificial systems and living organisms. Through the integration of mechanical compliance, environmental adaptability, and modular functionality, these systems are rapidly evolving to address the needs of next-generation biomedical, wearable, and environmental technologies. This review summarizes recent advances in soft robotics from the perspectives of material innovation, structural programmability, functional synergy, and intelligent responsiveness. Representative platforms based on microneedle arrays and 4D-printed hydrogels exemplify how structural versatility can be coupled with application specificity, enabling multifunctional capabilities in transdermal delivery, wound healing, motion control, and biosensing regulation.

The emergence of multifunctional platforms has been largely driven by the rapid development of stimuli-responsive and bioinspired materials. Hydrogels, shape memory elastomers, protein composites, and liquid metals have expanded the library of deformable substrates capable of reversible transformation and adaptive feedback. These materials not only enable controlled shape morphing and energy conversion but also allow self-healing and feedback-driven actuation, mimicking the adaptive features of biological tissues. Combined with advanced multimaterial fabrication techniques such as 4D printing, microfluidic assembly, and direct ink writing, soft robotic systems now exhibit unprecedented levels of structure–function integration, supporting complex deformation, distributed sensing, and intelligent energy management.

Despite this progress, several key limitations continue to restrict real-world deployment. Long-term durability under cyclic deformation remains a central concern, particularly for biointegrated systems subjected to continuous mechanical stress, hydration fluctuations, and biological fouling. The relatively low output force and limited control precision of current soft actuators also hinder their capability for high-load operations or fine manipulation tasks. While compliance ensures safety and adaptability, it often compromises motion accuracy, load-bearing capacity, and response speed. Addressing these challenges requires the development of hybrid material systems that combine soft and rigid elements, as well as optimization of actuation efficiency through magnetic, pneumatic, or electrohydraulic enhancement strategies. In parallel, the realization of precise, closed-loop control must rely on high-resolution sensing networks, rapid feedback algorithms, and data-driven models capable of predicting nonlinear deformation and compensating in real time.

Power autonomy represents another critical barrier to progress. Many existing soft robotic systems depend on external power sources or tethered configurations, which limit their mobility and long-term usability. The integration of lightweight, stretchable energy modules such as triboelectric nanogenerators, microsupercapacitors, and biofuel cells provides a promising route toward untethered and self-sustained operation. In future designs, closed-loop power management strategies that couple energy harvesting, storage, and redistribution with system-level control will be essential for achieving stable, continuous performance under dynamic environmental conditions.

A major technological frontier lies in the convergence of materials science, mechanical design, and artificial intelligence. The next generation of soft robots is expected to move beyond reactive functionality toward true autonomy through the incorporation of embedded logic, soft memory, and neuromorphic computing architectures. Machine learning algorithms and bioinspired control frameworks can enable mechanical intelligence, allowing soft systems to perceive stimuli, learn from deformation histories, and adaptively reprogram their behaviors. Such capabilities require the co-design of soft hardware and data-driven control models, ensuring compatibility between deformable substrates and real-time computation. Meanwhile, modular and reconfigurable system architectures will facilitate scalable manufacturing, targeted repair, and task-specific customization, establishing a foundation for complex, multifunctional operation.

Looking forward, the advancement of soft robotics depends on establishing a coherent technological roadmap that connects material robustness, structural adaptability, and intelligent control within a unified design philosophy. Future research should prioritize the creation of durable, fatigue-resistant, and biocompatible materials capable of long-term operation in biomedical and environmental contexts; the realization of hybrid architectures that balance compliance with force output and precision; and the integration of distributed energy networks for autonomous operation. At the same time, the development of intelligent algorithms, embedded sensors, and learning-based controllers will transform soft robots from passive responders into self-evolving systems capable of prediction and decision-making. These efforts require an interdisciplinary framework that links materials chemistry, mechanical engineering, and computational intelligence in a continuous feedback loop. By fostering such cross-domain collaboration, soft robotics can progress from isolated demonstrations to functional, adaptive platforms with tangible impact in healthcare, environmental monitoring, and human–machine interaction. Ultimately, the pursuit of seamless synergy among matter, mechanics, and intelligence will define the future landscape of this field and guide the realization of truly autonomous, life-mimicking robotic systems.

## Supplementary Information

Below is the link to the electronic supplementary material.

Supplementary file1 (DOCX 1085 KB)
