# Supplementary material for: Microscale Architectures for Intelligent Soft Robotics: From Functional Microneedles to Biointegrated Wearable Systems
Source: Nanomicro Lett. 2026 Jan 5;18:179. doi: 10.1007/s40820-025-02026-2 (PMC12765782; doi:10.1007/s40820-025-02026-2)
Supplement: Supplementary file 1 — Supplementary file1 (DOCX 1085 KB) [file 40820_2025_2026_MOESM1_ESM.docx]

Supporting Information for

**Microscale Architectures for Intelligent Soft Robotics: From Functional Microneedles to Biointegrated Wearable Systems**

Xin Li^1^, Ran Xu^2^, Chenchen Xie^2^, Zhixing Ge^3,*^, Bingbing Gao^2,3,*^ and Chwee Teck Lim ^3,4,5^

^1^ College of Biotechnology and Pharmaceutical Engineering, Nanjing Tech University, Nanjing 211816, P. R. China

^2^ School of Pharmaceutical Sciences, Nanjing Tech University, Nanjing 211816, P. R. China

^3^ Institute for Health Innovation and Technology (iHealthtech), National University of Singapore, Singapore 117599, Singapore

^4^ Department of Biomedical Engineering, National University of Singapore, 117583, Singapore

^5^ Mechanobiology Institute, National University of Singapore, 117411, Singapore

*Corresponding authors. E-mail: [gzx-01@nus.edu.sg](mailto:gzx-01@nus.edu.sg) (Zhixing Ge); [gaobb@njtech.edu.cn](mailto:gaobb@njtech.edu.cn) (Bingbing Gao)

# **S1 Structure of Soft Robots**

## **S1.1** **Microneedle Array Structures**

### ***S1.1.1 Solid MNs***

The application of solid microneedle structures in the field of soft robotics is expanding rapidly, particularly in terms of enhancing the perception and interaction capabilities of robots. Microarray microneedle tactile sensors (MTSs) used for intelligent object recognition employ spike-like microstructures based on silica gel. [S1] This solid microneedle array provides soft robots with precise tactile information regarding the shape, size, and texture of objects. Its miniature size of just 6 millimeters and exceptional shape adaptability enable robots to identify and perceive curved materials effectively. When combined with machine learning technology, this approach significantly enhances a robot's intelligent recognition capabilities, thereby facilitating the development of soft robot systems with autonomous operation and multimodal perception capabilities. Additionally, although the research focuses differ, the robust structural characteristics of conical microneedle arrays used in multifunctional microneedle patches also provide insights for functional integration in soft robots. These solid microneedles can maintain structural integrity while combining with materials such as carbon nanotubes and hydrogels to achieve flexibility and multifunctionality. For example, their demonstrated capabilities in drug delivery and pressure in the biomedical field suggest their potential as controllable actuators or sensing interfaces in soft robots. (Fig. S1a) [S2]

### ***1.1.2 Hollow MNs***

Hollow microneedle structures have demonstrated significant application potential in the field of soft robotics, particularly in integrating biosensing, microfluidic manipulation, and material delivery functions. (Fig. S1b) [S3, S4] For example, hollow microneedle arrays based on soft hollow microfibers have been developed as stretchable microfluidic biosensing patches capable of integrated blood sampling and electrochemical biosensing functions. This soft microneedle array, composed of flexible hollow microfibers, is manufactured using nanocomposite materials (containing polyimide, polyvinylidene fluoride‒trifluoroethylene copolymer, and single-walled carbon nanotubes) and nickel‒gold plating to increase the mechanical strength and biocompatibility. It can effectively collect bodily fluids via negative pressure and transport them to flexible electrochemical biosensors for precise detection, such as glucose detection. This design emphasizes the integration of soft materials, stretchable microfluidic devices, and flexible biosensors, enabling conformable contact with soft skin and laying the foundation for future wearable, minimally invasive biomolecular detection self-testing systems. Additionally, advancements in adjustable hollow polymer microneedles have opened new possibilities for soft robotics. These MNs optimize drug loading and control drug release kinetics by adjusting the cavity volume, aiming to overcome challenges such as insufficient drug loading and sustained release issues in traditional drug delivery. Although primarily applied in drug sustained release for diabetes treatment, their adjustable hollow structure, excellent mechanical strength, and potential for achieving multidose or diversified drug release kinetics make them useful in soft robotics as integrated fluid actuators, precise chemical delivery systems, or responsive material platforms. These studies collectively demonstrate that hollow microneedles, with their unique internal channels and controllable flexible properties, are driving advancements in soft robotics across complex environmental sensing, intelligent interactions, and multifunctional integration.

### ***1.1.3 Porous MNs***

Porous microneedles (Porous MNs) exhibit unique advantages in the field of soft robotics, particularly in achieving intelligent response and controlled substance delivery. For example, Gholami studied dynamically sealed hierarchical porous microneedles whose core lies in using mesoporous silica (SiO₂) to construct microneedles with hierarchical pore structures and then sealed the surface with a chitosan hydrogel to form a pH-responsive “nanovalve”. This design enables microneedles to be loaded with drugs after manufacturing and to achieve self-regulated transdermal delivery of insulin in response to glucose levels. [S5] This “glucose-responsive” and “self-regulated” mechanism holds significant implications for soft robotics. This finding suggests that porous microneedles can serve as a platform for integrating smart materials, enabling soft robots to sense specific environmental stimuli (such as biological signals or chemical concentration changes) and trigger precise substance release (such as drugs or chemical reagents) or drive adaptive deformation of flexible actuators. This smart response capability based on porous microneedles holds promise for advancing soft robots toward more advanced and personalized functions in fields such as biomedicine, environmental monitoring, and intelligent interaction [S6].

### ***1.1.4 Inverse Opal MNs***

Inverse opal microneedles (inverse opal MNs) exhibit unique and significant application potential in the field of soft robotics, particularly in the development of intelligent biointerfaces with high-sensitivity biosensing and real-time diagnostic capabilities. These MN arrays are fabricated via a colloidal crystal template method, resulting in highly ordered and interconnected porous structures that significantly increase the surface area and optical path length, thereby markedly enhancing the fluorescence signals. [S7] For example, inverse opal microneedle arrays have been developed for the in situ extraction of skin interstitial fluid biomarkers and fluorescence-enhanced screening. This system can effectively and sensitively detect bacterial infections at an early stage, achieving precise capture and analysis of biomarkers through its unique optical properties and microfluidic transport capabilities. Although this research focused primarily on medical diagnostics, the highly efficient biofluid extraction capabilities, fluorescence enhancement mechanisms, and high-sensitivity detection of trace biomarkers demonstrated in this study have profound implications for soft robots. This means that soft robots can utilize inverted opal microneedle arrays as part of their flexible bodies or end effectors to achieve noninvasive or minimally invasive real-time monitoring of the environment or internal biological systems. For example, in medical assistance robots, smart wearable diagnostic devices, or biomimetic robots with adaptive sensing capabilities, this technology can provide unprecedented diagnostic accuracy and response speed. Therefore, inverted opal microneedle structures offer an innovative solution for integrating advanced biosensing and intelligent diagnostic functions into soft robots.

## **1.2 Multilayer Composite Structures**

### ***1.2.1 Sandwich-like materials***

Sandwich-like structures in soft robotics, particularly in biomedical soft robotics, offer a new design paradigm for achieving multifunctional integration and complex environmental interactions.[S8] A typical example is the integrated conductive microneedle patch for treating myocardial infarction (MI) via induced cardiac muscle cells (iPSC-CMs), whose core is a sophisticated sandwich-like structure. The patch consists of three layers: the bottom layer is a drug-encapsulated microneedle layer responsible for local drug delivery; the middle layer is a conductive layer composed of parallel carbon nanotubes (CNTs), providing the necessary electrical signal conduction pathways; and the top layer is a methyl acrylate-modified gelatin (GelMA) hydrogel scaffold used to support and integrate induced pluripotent stem cell-derived cardiomyocytes. This multilayer design enables the patch to perform multiple functions synergistically, including drug delivery, electrical signal conduction, and cell integration, to promote tissue regeneration. Its ability to integrate multimaterial, multifunctional components on a flexible substrate, coupled with its ability to regulate complex biological processes through the synergistic interaction of its layers, aligns closely with the demands of soft robotics for highly integrated, adaptive, and biocompatible interfaces. The design philosophy of this sandwich structure provides important insights for developing flexible systems with multiple functions, such as sensing, actuation, therapy, or diagnosis, in soft robotics, enabling robots to interact with biological organisms more precisely and safely.

### ***1.2.2 Cap-Doffing System***

The “Cap-Doffing System” demonstrates significant potential in soft robotics, particularly in biointegrated robots that require intelligent response and precise material release. These systems enable soft robots to achieve self-regulating functionality through dynamic capping mechanisms. A typical example is the layered porous microneedle used for insulin self-regulating transdermal delivery, whose core is a clever dynamic “cap-doffing system”. [S5] This system utilizes the nanopores of mesoporous silica (MS) and employs an enzyme-loaded chitosan (CS) hydrogel as a dynamic cap. When exposed to specific stimuli, such as changes in glucose concentration, enzymatic reactions cause the CS hydrogel to undergo reversible swelling or deswelling, thereby ‘opening’ or “closing” the cap and precisely controlling the on-demand release of encapsulated substances. This mechanism not only provides ‘on/off’ drug release performance but also demonstrates rapid and repeatable responses to physiological stimuli. For soft robots, this ‘cap-removal system’ capable of adaptively releasing substances or regulating channels in response to environmental changes opens up broad prospects for applications in drug delivery, chemical sensing, self-healing material integration, and the development of intelligent soft actuators with complex biomimetic functions, enabling them to interact more intelligently and precisely with complex environments.

### ***1.2.3 Separable-Tip System***

The separable-tip system represents a highly intelligent and practical design paradigm in soft robotics, particularly in the field of micromedical robots, aimed at optimizing drug delivery within the body while ensuring biosafety. [S9] This system achieves precise delivery and safe removal by separating the functional and control components of the microneedle through a sophisticated structural design. For example, to achieve efficient oral delivery of large-molecule drugs to the intestines, researchers have developed a magnetically responsive MN robot whose core is a separable tip system composed of three key components: a magnetic base, a separable connector, and a drug-carrying tip. The MN robot was fabricated via an LEGO-like multistage 3D manufacturing strategy. After oral administration, it enters the small intestine protected by an enteric capsule and releases the drug into the intestinal environment. Owing to its polarized magnetic base, the microneedle tip can be guided by a specific magnetic field to target the small intestinal wall, penetrate the complex gastrointestinal barrier, and implant itself into the tissue to deliver the encapsulated active substance. The unique feature of this system is that once the detachable connector degrades, the drug-carrying tip remains in the tissue to continuously release the active substance, while the magnetic base is safely excreted from the body. This design not only significantly improves the oral bioavailability of large-molecule drugs (such as insulin), addressing the challenges of traditional oral administration but also ensures long-term efficacy and ultimate biosafety through an intelligent separation mechanism, opening a new chapter in the development of novel, efficient, and safe soft robots for in vivo drug delivery.

## **1.3** **Smart-actuated architectures**

### ***1.3.1Magnetic Microneedle Robots***

Magnetic microneedle robots, as cutting-edge developments in the field of soft robotics, have opened new avenues for precise manipulation and remote intervention in biomedical applications [S10–S12]. These robots utilize external magnetic fields for noncontact control, enabling targeted delivery or precise manipulation within the body. For example, to overcome intestinal barriers and achieve efficient oral delivery of large-molecule drugs, researchers have developed a magnetic MN robot. The robot is constructed via a modular, multistage 3D printing strategy and consists of three components: a magnetic base, a detachable connector, and a drug-loaded tip. After oral administration, under the influence of a specific magnetic field, the polarized magnetic base guides the tip to orient and insert into the small intestinal wall, enabling precise delivery of active substances such as insulin. The detachable connector subsequently degrades, allowing the tip to release the drug continuously, while the magnetic base is safely expelled from the body, significantly increasing drug bioavailability and safety. Furthermore, the layered magnetic MN array robot has also been applied to controlled tissue cutting (histotomy). This robot can precisely cut primary tissue to establish high-throughput organoid-on-a-chip models, thereby addressing the shortcomings of traditional tissue sectioning and culture techniques in meeting clinical needs. These studies collectively demonstrate that magnetically responsive MN robots, owing to their externally controllable motion, precise positioning capabilities, and multifunctional integration characteristics, exhibit tremendous potential in soft robotics applications such as smart drug delivery, minimally invasive surgical assistance, and advanced bioengineering platform construction. [S9, S13]

### ***1.3.2 Electroactive System***

Electroactive systems are playing an increasingly important role in the field of soft robotics, particularly in enhancing their sensory capabilities and intelligent interaction. These systems can convert mechanical stimuli into electrical signals or generate mechanical responses through electrical stimulation, thereby endowing robots with environmental perception and adaptive functions. A microneedle tactile sensor (MTS) for intelligent object recognition operates on the basis of the triboelectric effect and electrostatic induction. The sensor is constructed using a silicone-based composite material, enabling it to efficiently convert intuitive information such as the shape, size, and texture of an object during contact into electrical signals, thereby assisting robots in making more precise decisions. This miniaturized electroactive tactile sensor not only exhibits excellent shape adaptability and is capable of identifying curved surfaces but also significantly enhances a robot's object recognition capabilities when combined with machine learning algorithms. This design concept demonstrates how electroactive systems can simulate the tactile perception capabilities of biological organisms through precise material and structural design, enabling soft robots to interact with complex environments in a refined manner. It also lays the technological foundation for future advanced soft robot systems with autonomous operation and multimodal perception capabilities. [S2]

### ***1.3.3 Optogenetic-Triggered MNs***

Optogenetically triggered MNs (optogenetically triggered MNs) represent a cutting-edge integrated technology in the field of biomedical soft robotics. By combining optogenetic tools with microneedle delivery systems, they enable precise, remote regulation of biological processes within the body. These systems allow researchers or robots to manipulate cellular activity or substance release with high specificity and temporal resolution via light signals. A self-powered triboelectric-responsive microneedle system designed for intervertebral disc degeneration (IVDD) repair. This microneedle array can controllably release optogenetically engineered extracellular vesicles (EVs). These EVs achieve sustainable release through the triboelectric effect, and the TRAMI protein loaded within them is also functionalised via optogenetics, enabling them to target and precisely regulate the intracellular DNA-sensing pathways associated with inflammatory activation in aged nucleus pulposus cells. This design allows the microneedle system to deliver light-sensitive or light-regulated bioactive substances, thereby enabling remote, noninvasive, and high-precision intervention in complex biological pathological processes. The core of the microneedle lies in delivering and activating optogenetic functional elements, endowing soft robots with the ability to simulate complex biological interactions. This provides a new paradigm for future targeted therapy, intelligent diagnosis, and the development of biorobots deeply integrated with living tissues. [S14]

## **1.4** **Flexible and Stretchable architectures**

### ***1.4.1 Flexible MN Electrodes***

Flexible microneedle electrodes (Flexible MN Electrodes) are a key technology in the field of soft robotics, enabling robots to achieve seamless, conformable electrical interactions with biological tissues [S15–S17]. This technology holds significant potential in areas such as biosensing, neural interfaces, and tissue engineering. These electrodes typically combine minimally invasive penetration capabilities with material flexibility to adapt to irregular biological surfaces while ensuring effective transmission or collection of electrical signals. Hollow microneedle arrays based on soft microfibers have been developed as stretchable microfluidic biosensing patches that integrate flexible electrochemical biosensors. This system utilizes hollow microneedle arrays manufactured from flexible hollow microfibers, which are imparted with conductivity through nickel‒gold plating, enabling minimally invasive sampling from interstitial fluid and precise biomolecular detection. This provides an ideal flexible electrode solution for wearable biosensing and diagnostic soft robots. Additionally, an inductively integrated conductive microneedle patch for treating myocardial infarction (MI) demonstrates the application of flexible microneedle electrodes. The patch features parallel carbon nanotube (CNT) conductive intermediate layers, providing electrical conduction pathways, thereby imparting flexibility and conductivity to the entire patch. This enables the transmission of electrophysiological signals and the regulation of cellular activity, further supporting tissue regeneration. These microneedle electrodes combine conductive materials with flexible substrates, minimize tissue damage, enhance signal stability, and improve long-term wear comfort, providing critical technical support for soft robotics in long-term monitoring, stimulation, or treatment within biological organisms. [S1, S4]

### ***1.4.2 Stretchable microfluidic platform***

Stretchable microfluidic platforms represent a significant advancement in the fields of soft robotics and wearable biomedical devices [S18, S19]. By combining flexible materials with microfluidic technology, these platforms achieve conformal adhesion to irregular surfaces, such as human skin, while maintaining fluid transport and functional integrity under deformable conditions. The core advantage of such platforms lies in their inherent flexibility and extensibility, enabling them to overcome the limitations of traditional rigid devices and provide a more comfortable, stable, and nonintrusive biointerface. A stretchable microfluidic biosensing patch featuring an array of hollow microneedles made from soft microfibers. This platform integrates a stretchable microfluidic device, a flexible electrochemical biosensor, and a microneedle array made of flexible hollow microfibers. Through a negative pressure-driven sampling mechanism, the system can minimally invasively collect biological fluids from interstitial fluid and utilize flexible sensors for precise electrochemical detection of biomarkers (such as glucose). This design philosophy not only emphasizes the use of thin, soft materials but also highlights their ability to provide durable, nonirritating, stable, and user-friendly interfaces, which is crucial for developing next-generation wearable point-of-care testing (POCT) technology. Therefore, the stretchable microfluidic platform, with its unique physical properties and integrated multifunctionality, significantly expands the application scope of soft robotics in fields such as biomonitoring, drug delivery, and smart diagnostics, enabling it to interact more effectively with complex and dynamic biological systems. [S20]

## **1.5** **Bioinspired and Biomimetic Design**

### ***1.5.1 Imitation plant spiny structure***

Bioinspired plant-like spiny structures demonstrate exceptional design ingenuity in the fields of biointegrated soft robots and wearable devices. Drawing inspiration from the morphology and functionality of natural plant spines, these structures enable optimized tissue penetration, fixation, and seamless integration with dynamic biological interfaces. This biomimetic design significantly enhances device stability and biocompatibility, making it particularly suitable for applications requiring long-term, high-precision monitoring. Inspired by plant spines, the skin-integrated, biocompatible, and stretchable silicon microneedle electrode (SSME) is specifically designed for long-term electromyography (EMG) monitoring in motion scenarios. The electrode adopts a silicon microneedle structure and is cleverly semiencapsulated with polyimide (PI). This composite structure not only enhances the electrode's adaptability to deformation and fatigue resistance but also provides it with a stretchability of no less than 36%. The plant-thorn-inspired SSME was prepared via a semiadditive method, and its biocompatibility was verified through cytotoxicity testing. This design enables the electrode to securely and comfortably adhere to the skin surface, enabling accurate and stable EMG signal acquisition even during human movement. This provides an innovative solution for neuromuscular function assessment and efficient, reliable human‒machine interfaces. Owing to its unique mechanical properties and compatibility with biological tissues, the plant-inspired spiny structure clearly offers important biomimetic design insights for developing the next generation of intelligent, flexible, and long-lasting biointegrated soft systems. (Fig. S1c) [S21]

### ***1.5.2 Fish-like adsorption structure***

Fish-inspired adhesive structures provide important biomimetic examples for the field of soft robotics, particularly for robot designs that require both strong adhesion and efficient mobility. These structures draw inspiration from fish, especially climbing fish, which exhibit unique adhesion and sliding mechanisms in complex, moist environments. By uncovering the secret behind climbing fish's ability to maintain tight adhesion while achieving rapid sliding, research has revealed that the key lies in the synergistic interaction between the flexible ‘lamellar structures’ on their adhesion discs and the hardened ‘epidermal spines.’ The flexible lamellae primarily provide wet adhesion or suction cup effects, ensuring close contact with the substrate, whereas the hardened spines provide additional friction or anchoring during sliding, thereby balancing adhesion strength and movement efficiency. This ingenious biological design points the way for the development of bioinspired robots, enabling them to replicate or surpass the movement and manipulation capabilities of natural organisms on complex surfaces. Integrating this fish-inspired adhesion structure into soft robots holds promise for unprecedented adhesion and mobility performance in fields such as underwater exploration, gripping on wet surfaces, and high-adaptability interactions with unstructured environments. (Fig. S1d) [S22]

## **1.6 Textile Structure**

Advanced textile architectures for soft robots achieve high-performance, multi-modal flexible actuation by integrating active materials, such as liquid crystal elastomers (LCEs), with custom-designed textile topologies. Specifically, LCE actuators based on knitting technology utilize loop structures and internal torque to transform the contraction of LCE fibers into quantitatively controllable, complex 3D deformations, simultaneously exhibiting excellent processability and recyclability [S23]. Furthermore, bioinspired hierarchical textile-structured pneumatic actuators ingeniously combine dual-stiffness boucle fancy yarns with a novel trilayer-knit architecture. This design imparts super-anisotropic mechanical properties to the actuators, providing advantages such as high power density and fast response at low operating pressures, making them particularly suitable for wearable healthcare devices (Fig. S1e) [S24]. These advanced fabrication strategies collectively drive the development of soft robotics toward complex deformation, functional integration, and commercial viability.


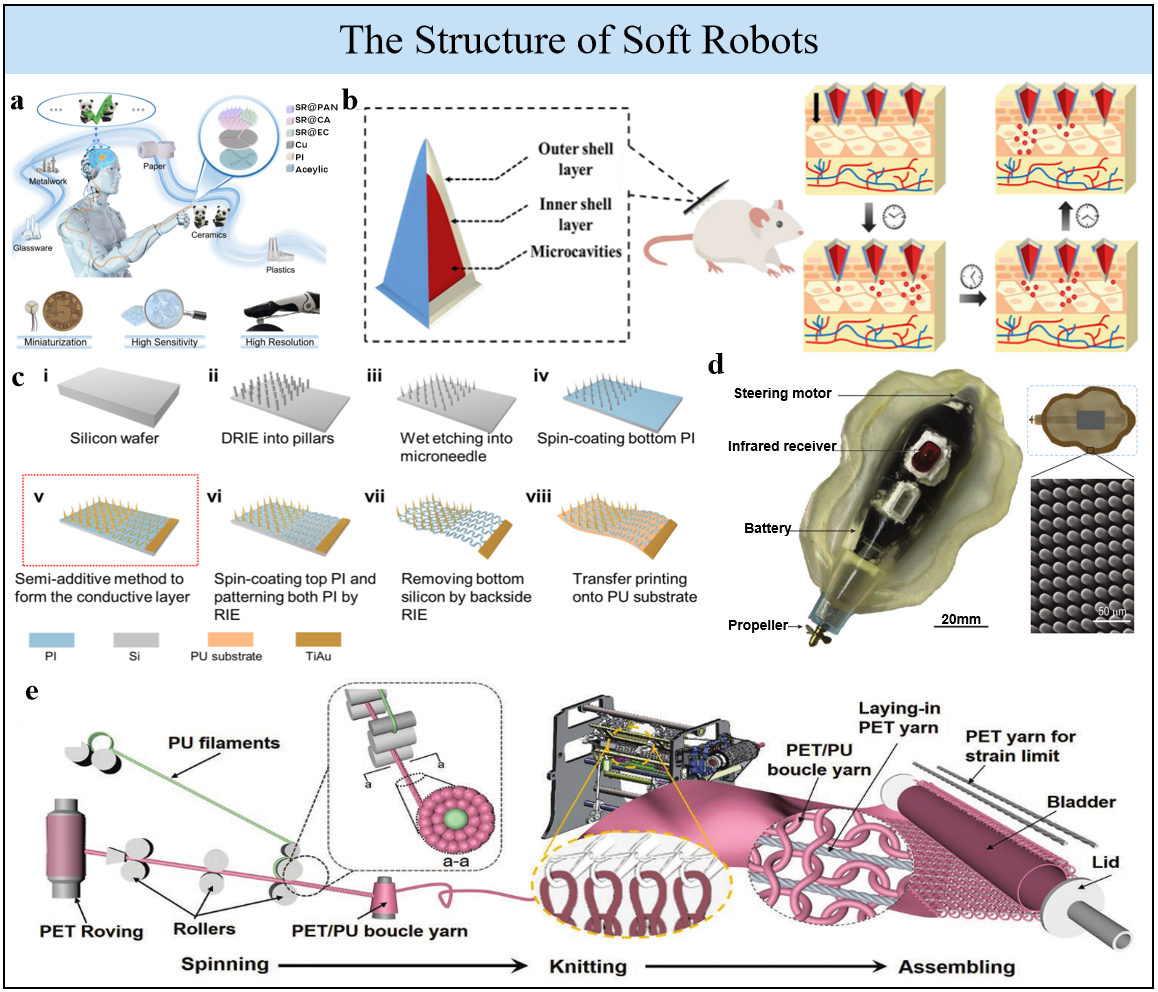


**Fig. S1** **a** Solid Microneedle Design for Miniature Sensors in Intelligent Object Recognition. [S2] Copyright 2024 Elsevier **b** Schematic Diagram of the Long-Term Hypoglycemic Effect of Hollow Adjustable Microneedles on Diabetic Rats. [S3] Copyright 2024 Elsevier **c** Process Flow for Fabricating Microfluidic Channels and Microneedles in SMNEA Devices. [S21] Copyright 2023 Springer Nature **d** Schematic Diagram of the Rock-Climbing Fish Robot Structure. [S22] Copyright 2023 Oxford University Press **e** Schematic Diagram of the Layered Manufacturing Process for Structural Actuators. [S24] Copyright 2023 Wiley-VCH

**Supplementary References**

1. L. Sun, Y. Wang, L. Fan, Y. Zhao, Multifunctional microneedle patches with aligned carbon nanotube sheet basement for promoting wound healing. Chem. Eng. J. **457**, 141206 (2023). <https://doi.org/10.1016/j.cej.2022.141206>
2. Z. An, Z. Wu, Y. Hu, C. Han, Z. Cao et al., A miniaturized array microneedle tactile sensor for intelligent object recognition. Nano Energy **125**, 109567 (2024). <https://doi.org/10.1016/j.nanoen.2024.109567>
3. T. Liu, Y. Sun, W. Zhang, R. Wang, X. Lv et al., Hollow-adjustable polymer microneedles for prolonged hypoglycemic effect on diabetic rats. Chem. Eng. J. **481**, 148670 (2024). <https://doi.org/10.1016/j.cej.2024.148670>
4. M.V. Chinnamani, A. Hanif, P.K. Kannan, S. Kaushal, M.J. Sultan et al., Soft microfiber-based hollow microneedle array for stretchable microfluidic biosensing patch with negative pressure-driven sampling. Biosens. Bioelectron. **237**, 115468 (2023). <https://doi.org/10.1016/j.bios.2023.115468>
5. S. Gholami, I. Zarkesh, M.-H. Ghanian, E. Hajizadeh-Saffar, F. Hassan-Aghaei et al., Dynamically capped hierarchically porous microneedles enable post-fabrication loading and self-regulated transdermal delivery of insulin. Chem. Eng. J. **421**, 127823 (2021). <https://doi.org/10.1016/j.cej.2020.127823>
6. S. Kusama, K. Sato, Y. Matsui, N. Kimura, H. Abe et al., Transdermal electroosmotic flow generated by a porous microneedle array patch. Nat. Commun. **12**, 658 (2021). <https://doi.org/10.1038/s41467-021-20948-4>
7. K. Yi, Y. Yu, Y. Wang, Y. Zhao, Inverse opal microneedles arrays for fluorescence enhanced screening skin interstitial fluid biomarkers. Nano Today **47**, 101655 (2022). <https://doi.org/10.1016/j.nantod.2022.101655>
8. L. Sun, X. Zhu, X. Zhang, G. Chen, F. Bian et al., Induced cardiomyocytes-integrated conductive microneedle patch for treating myocardial infarction. Chem. Eng. J. **414**, 128723 (2021). <https://doi.org/10.1016/j.cej.2021.128723>
9. X. Zhang, G. Chen, X. Fu, Y. Wang, Y. Zhao, Magneto-responsive microneedle robots for intestinal macromolecule delivery. Adv. Mater. **33**(44), e2104932 (2021). <https://doi.org/10.1002/adma.202104932>
10. M. Seong, K. Sun, S. Kim, H. Kwon, S.-W. Lee et al., Multifunctional magnetic muscles for soft robotics. Nat. Commun. **15**, 7929 (2024). <https://doi.org/10.1038/s41467-024-52347-w>
11. Z. Chen, Y. Wang, H. Chen, J. Law, H. Pu et al., A magnetic multi-layer soft robot for on-demand targeted adhesion. Nat. Commun. **15**, 644 (2024). <https://doi.org/10.1038/s41467-024-44995-9>
12. P. Yang, L. Mao, C. Tian, X. Meng, H. Xie, A cooperative and multifunctional magnetic continuum robot for noninteractive access, dexterous navigation, and versatile manipulation. Adv. Funct. Mater. **35**(2), 2412543 (2025). <https://doi.org/10.1002/adfm.202412543>
13. X. Zhang, H. Chen, T. Song, J. Wang, Y. Zhao, Controllable histotomy based on hierarchical magnetic microneedle array robots. Engineering **42**, 166–174 (2024). <https://doi.org/10.1016/j.eng.2024.05.004>
14. W. Zhang, X. Qin, G. Li, X. Zhou, H. Li et al., Self-powered triboelectric-responsive microneedles with controllable release of optogenetically engineered extracellular vesicles for intervertebral disc degeneration repair. Nat. Commun. **15**(1), 5736 (2024). <https://doi.org/10.1038/s41467-024-50045-1>
15. Y. Zhao, S. Shen, R. Cao, H. Wu, H. Yu et al., Flexible stretchable tribo-negative films with exceptional output performance for high-temperature energy harvesting and self-powered sensor. Nano Energy **114**, 108654 (2023). <https://doi.org/10.1016/j.nanoen.2023.108654>
16. K. Veenuttranon, K. Kaewpradub, I. Jeerapan, Screen-printable functional nanomaterials for flexible and wearable single-enzyme-based energy-harvesting and self-powered biosensing devices. Nano-Micro Lett. **15**, 85 (2023). <https://doi.org/10.1007/s40820-023-01045-1>
17. J. Lyu, Q. Zhou, H. Wang, Q. Xiao, Z. Qiang et al., Mechanically strong, freeze-resistant, and ionically conductive organohydrogels for flexible strain sensors and batteries. Adv. Sci. **10**(9), 2206591 (2023). <https://doi.org/10.1002/advs.202206591>
18. H. Kim, J. Lee, U. Heo, D.K. Jayashankar, K.-C. Agno et al., Skin preparation-free, stretchable microneedle adhesive patches for reliable electrophysiological sensing and exoskeleton robot control. Sci. Adv. **10**(3), eadk5260 (2024). <https://doi.org/10.1126/sciadv.adk5260>
19. M. Park, T. Park, S. Park, S.J. Yoon, S.H. Koo et al., Stretchable glove for accurate and robust hand pose reconstruction based on comprehensive motion data. Nat. Commun. **15**, 5821 (2024). <https://doi.org/10.1038/s41467-024-50101-w>
20. Q. Zhao, E. Gribkova, Y. Shen, J. Cui, N. Naughton et al., Highly stretchable and customizable microneedle electrode arrays for intramuscular electromyography. Sci. Adv. **10**(18), eadn7202 (2024). <https://doi.org/10.1126/sciadv.adn7202>
21. H. Ji, M. Wang, Y. Wang, Z. Wang, Y. Ma et al., Skin-integrated, biocompatible, and stretchable silicon microneedle electrode for long-term EMG monitoring in motion scenario. NPJ Flex. Electron. **7**, 46 (2023). <https://doi.org/10.1038/s41528-023-00279-8>
22. W. Tan, C. Zhang, R. Wang, Y. Fu, Q. Chen et al., Uncover rock-climbing fish’s secret of balancing tight adhesion and fast sliding for bioinspired robots. Natl. Sci. Rev. **10**(8), nwad183 (2023). <https://doi.org/10.1093/nsr/nwad183>
23. J. Sun, W. Liao, Z. Yang, Additive manufacturing of liquid crystal elastomer actuators based on knitting technology. Adv. Mater. **35**(36), e2302706 (2023). <https://doi.org/10.1002/adma.202302706>
24. M. Yang, J. Wu, W. Jiang, X. Hu, M.I. Iqbal et al., Bioinspired and hierarchically textile-structured soft actuators for healthcare wearables. Adv. Funct. Mater. **33**(5), 2210351 (2023). <https://doi.org/10.1002/adfm.202210351>
